# Identification of Novel PFAS Transformation Products in AFFF-Impacted Surface and Groundwater

**DOI:** 10.1021/acs.est.6c11616

**Published:** 2026-10-14

**Authors:** Emmanuel C. Tolefe, Gavyn C. Lastoria, Jaanus Liigand, Lindsay S. Cahill, Karl J. Jobst

**Affiliations:** † Department of Chemistry, 7512 Memorial University of Newfoundland, 45 Arctic Avenue, St. John’s NL A1C 5S7, Canada; ‡ Quantem Analytics, 51008 Tartu, Estonia

**Keywords:** non-targeted screening, suspect screening, unregulated PFAS, transformation products, environmental fate, ion mobility, high resolution mass spectrometry, chemical fingerprinting, multidimensional separation, drinking water

## Abstract

The widespread use of Aqueous Film-Forming Foams (AFFF) has left thousands of contaminated “hotspots” across North America. In Canada, only 25 per-/polyfluoroalkyl substances (PFAS) are regulated in drinking water at 30 ng/L. In the present study, cyclic ion mobility–tandem mass spectrometry (cIMS-MS^2^) was used to identify 17 regulated and 52 unregulated PFAS in surface waters near a former firefighting training facility in Atlantic Canada, including 6 new PFAS that have not been previously reported in environmental waters: hydrido-ketone-5:2-fluorotelomer sulfonate (FTS), ketone-8:2-FTS, unsaturated 8:2-FTS, *N*-methyl-perfluorohexane sulfonamido sulfinate, *N*-methyl-perfluorooctane sulfonamido sulfinate, and perfluorohexane sulfonamido propionate. Semi-quantitation of unregulated PFAS was enabled by a machine-learning model validated using a set of 21 targeted PFAS (*R* ^2^ > 0.95). The total concentration of PFAS declined from 2500 ng/L to 364 ng/L as the surface water entered the North Atlantic approximately 5 km away from the hotspot. Seven of forty-two drinking water wells located along a 5 km transect from the source were characterized by exceedances between 30 and 191 ng/L. Twenty-seven PFAS were identified in the wells, including 13 that are unregulated and 5 that result from (bio)­transformation of AFFF. While the fraction of unregulated PFAS was low (<18%), these transformation products may inform forensic studies aimed at establishing links between the source of pollution and its receptors.

## Graphical abstract

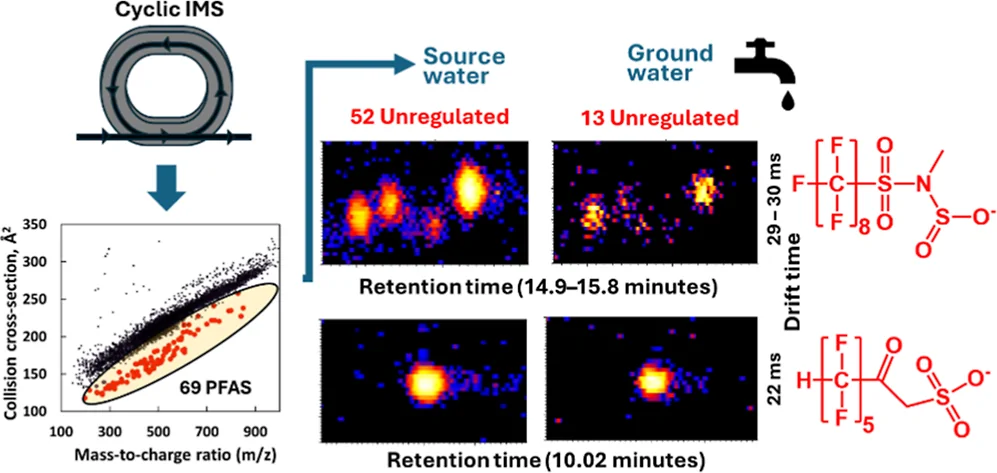

## 1 Introduction

Per- and polyfluoroalkyl substances (PFAS) are a structurally diverse class of synthetic compounds that share the –CF2- motif.^1^ Since the 1960s, they have been key ingredients in aqueous film-forming foams (AFFFs) used to fight fuel fires at airports, military bases, firefighter training facilities, and oil and gas infrastructure.^1,2^ The strong thermal and chemical stability afforded by the carbon–fluorine bond ensures that the foam blanket that suppresses the fire remains intact. However, this stability is also reflected by the persistence of PFAS in the environment.^3,4^ Legacy AFFF formulations predominantly consist of perfluorooctanesulfonates (PFOS) and perfluorohexanesulfonates (PFHxS)^5^ that are known to bioaccumulate in humans and wildlife. Animal exposure studies and epidemiological evidence show that exposure to these chemicals can cause adverse health effects at all stages of life, including metabolic disruption,^6^ immune suppression, pregnancy complications,^7,8^ and kidney and testicular cancer.^9^ Due to these concerns, production of long-chain PFAS was phased out in the early 2000s and replaced with less fluorinated, shorter-chain fluorotelomer (FT)-based alternatives.^10^ Approximately 2300 PFAS contamination sources are documented in Europe and North America, respectively, with over 100,000 suspected contamination cases.^11–13^

Environmental monitoring of AFFF-impacted sites have largely focused on a narrow set of PFAS, including legacy perfluorosulfonic acids (PFSAs) such as PFOS, their FT-based replacements, as well as perfluorocarboxylic acids (PFCAs), such as perfluorooctanoic acid (PFOA), that may be present as minor constituents in AFFF formulations,^14,15^ or produced as (bio)­transformation and degradation products. In Canada, a drinking water objective of 30 ng/L for the sum of 25 PFAS (both long- and short-chain) was recently established.^16^ In the United States, the Environmental Protection Agency has set enforceable limits on PFOA and PFOS at 4 ng/L, and the European Union’s new Drinking Water Directive sets a limit of 100 ng/L for the combined concentrations of 20 PFAS.^17,18^ However, the targeted analytical methods designed to support these regulations can overlook unregulated PFAS and potentially underestimate the exposure risk.

Early experiments using nuclear magnetic resonance (^19^F NMR) revealed the diversity of PFAS present in AFFFs.^19^ Pioneering studies by D’Agostino and Mabury^20^ and Barzen-Hanson et al.^21^ identified more than 50 additional (anionic and zwitterionic) PFAS classes in AFFFs. Once released into the environment, they undergo biotic and abiotic transformations, yielding persistent perfluoroalkyl acids (PFAAs) and intermediates.^21,22^ This has driven the development of non-targeted screening (NTS) strategies designed to identify undocumented PFAS and their (bio)­transformation products. In Canada, the Sauvé group has played a leading role in advancing the understanding of PFAS contamination at federal contamination sites.^23–25^ Liu et al.^25^ reported concentrations ranging from 800 ng/L to 3.7 million ng/L for the sum of 17 regulated PFAS at major Canadian airports, and identified more than 150 transformation products. Indeed, unregulated PFAS transformation products are increasingly being detected at contaminated sites in North America and Europe,^26–28^ and often arise from multiple sources such as landfill leachate, fluorochemical facilities, firefighter training, and municipal and industrial wastewater effluent, each contributing distinct chemical profiles.^29–32^ This complexity makes it difficult to establish clear links between the source of PFAS pollution and receptor populations, underlining the need to identify source-specific markers.

PFAS contamination from a firefighter training facility at a hotspot in Atlantic Canada, previously characterized by Liu et al.,^25^ is representative of AFFF-contaminated sites across North America. Groundwater concentrations near such sites have been reported to exceed Canadian and European guideline values by several orders of magnitude.^25,29,31,33^ The former firefighter training area (FFTA) has been inactive for over 20 years, and the absence of competing sources allows the contamination to be traced to a single, well-defined location. In the present study, we investigate the spread of PFAS into surface and groundwater adjacent to the hotspot using a novel experimental technique, viz. Cyclic ion mobility spectrometry (cIMS) coupled with tandem mass spectrometry (MS^2^).^34–36^ cIMS separates ions based on their movement through a buffer gas, which is governed by ion size, shape, and charge state, providing collision cross-section (CCS) values.^34,35^ cIMS-MS^2^ has been demonstrated as a PFAS discovery platform, enabling the separation of PFAS isomers^37^ and the identification of undocumented PFAS and their (bio)­transformation products by exploiting CCS differences, in addition to chromatographic retention time (RT) and MS resolution.^38–40^ The objectives of the present study include: (1) identifying previously undocumented PFAS and their (bio)­transformation products in contaminated surface water and groundwater using cIMS-MS^2^ experiments; (2) estimating exposure to both regulated and unregulated PFAS using authentic standards and a machine-learning model; (3) identifying transformation products of AFFF pollution that could potentially inform forensic studies aimed at linking the source with groundwater receptors.

## 2 Materials and Methods

### 2.1 Chemicals and Standards

HPLC-grade methanol (MeOH), ethyl acetate (EtAc), LCMS-grade water, ammonium acetate, formic acid (FA, 99%), and ACS-grade MeOH were obtained from Sigma-Aldrich (St. Louis, MO, USA) for sample preparation and chromatographic separation. Ammonium hydroxide (NH_4_OH, 28%) and sodium thiosulfate pentahydrate (Na_2_S_2_O_3_·5H_2_O) were purchased from ACP Chemicals Inc. (Montreal, QC, Canada). Native (EPA-533PAR), isotopically labeled (EPA-533ES), and injection (EPA-533IS) standards were obtained from Wellington Laboratories (Guelph, Ontario, Canada). Native PFAS (fully named in Table S1) included C_4_–C_12_ PFCAs (PFBA, PFPeA, PFHxA, PFHpA, PFOA, PFNA, PFDA, PFUdA, and PFDoA), C_4_–C_8_ PFSAs (PFBS, PFPeS, PFHxS, PFHpS, and PFOS), fluorotelomer sulfonates (4:2-, 6:2-, and 8:2 FTS), and emerging PFAS (PFEESA, NFDHA, ADONA, GenX, 9Cl-PF3ONS, and 11Cl-PF3OUdS). Internal standards included ^13^C_5_–PFPeA, ^13^C_5_–PFHxA, ^13^C_4_–PFHpA, ^13^C_8_–PFOA, ^13^C_9_–PFNA, ^13^C_6_–PFDA, ^13^C_7_–PFUdA,^13^C_1_–PFDoA,^13^C_3_–PFBS, ^13^C_3_–PFHxS, ^13^C_8_–PFOS, ^13^C_2_-4:2 FTS, ^13^C_2_-6:2 FTS, ^13^C_2_-8:2 FTS. ^13^C_2_–PFOA and ^13^C_4_–PFOS served as injection standards.

### 2.2 Sampling Sites, Sample Collection and Preparation

Sampling was conducted between December 2024 and November 2025 (sites shown in Figure 1a). It included six surface water sites. Sites SW1–SW4 were located downstream of the FFTA, while SW5 and SW6 were along an alternate distributary. Control surface water was obtained (one upstream and two downstream) from an adjacent catchment.^41^ Groundwater sampling consisted of 42 private wells (artesian and hand-dug) distributed across 4 sampling areas: area 1 (1.5 km; *n* = 10), area 2 (2.5 km; *n* = 12), area 3 (3.5 km; *n* = 16), and area 4 (4.5 km; *n* = 4); and control wells (*n* = 10). Private well owners provided written informed consent before participating in the study, which was approved by the Newfoundland and Labrador Health Research Ethics Authority (Approval No. HREB # 2024.222). Samples and field blanks (*n* = 8) were collected in 1 L HDPE bottles pre-rinsed with MeOH. On-site, 156 mg of Na_2_S_2_O_3_·5H_2_O was added, and samples were stored at 4 °C. Holding times did not exceed 30 days. Following gravity settling of suspended particulates, aliquots (500 mL) of the stored samples and blanks were spiked with internal standard (10 μL of 25–100 pg/μL EPA533ES prepared in MeOH), and adjusted to pH 6.3 ± 0.1 using 0.5% FA and NH_4_OH. Samples were extracted without filtration to represent whole-water PFAS concentrations and avoid losses associated with filtration.^42^ Extraction was performed by solid-phase extraction (SPE) using a weak anion-exchange cartridge (Waters Oasis WAX 6cc, 150 mg/30 μm). SPE cartridges were conditioned using 8 mL of 0.1% NH_4_OH in MeOH/EtAc (1:1), 8 mL MeOH/EtAc (1:1), and 4 mL of LCMS-grade water. The samples were loaded onto the cartridges under vacuum at a rate of 1∼2 drops/second, washed with ammonium acetate buffer (pH 7.5), dried under vacuum for 10 min, and eluted into 15 mL polypropylene (PP) centrifuge tubes (Ultident Inc., Progene, sterile) using 12 mL of 0.1% NH_4_OH in MeOH/EtAc (1:1). Eluates were concentrated to 0.5 mL at 30 ^o^C in a centrifugal vacuum concentrator (CentriVap, Labconco, USA) and pipetted into 2 mL PP vials. To improve recovery, the inner walls of the tubes were vortex-rinsed with 0.5 mL MeOH, which was also added to the vials and reconcentrated. The extracts were spiked with injection standard (10 μL of 100–300 pg/μL EPA533IS in MeOH) and made up to 625 μL (80:20 MeOH/LCMS-grade H_2_O).

**Figure 1:**
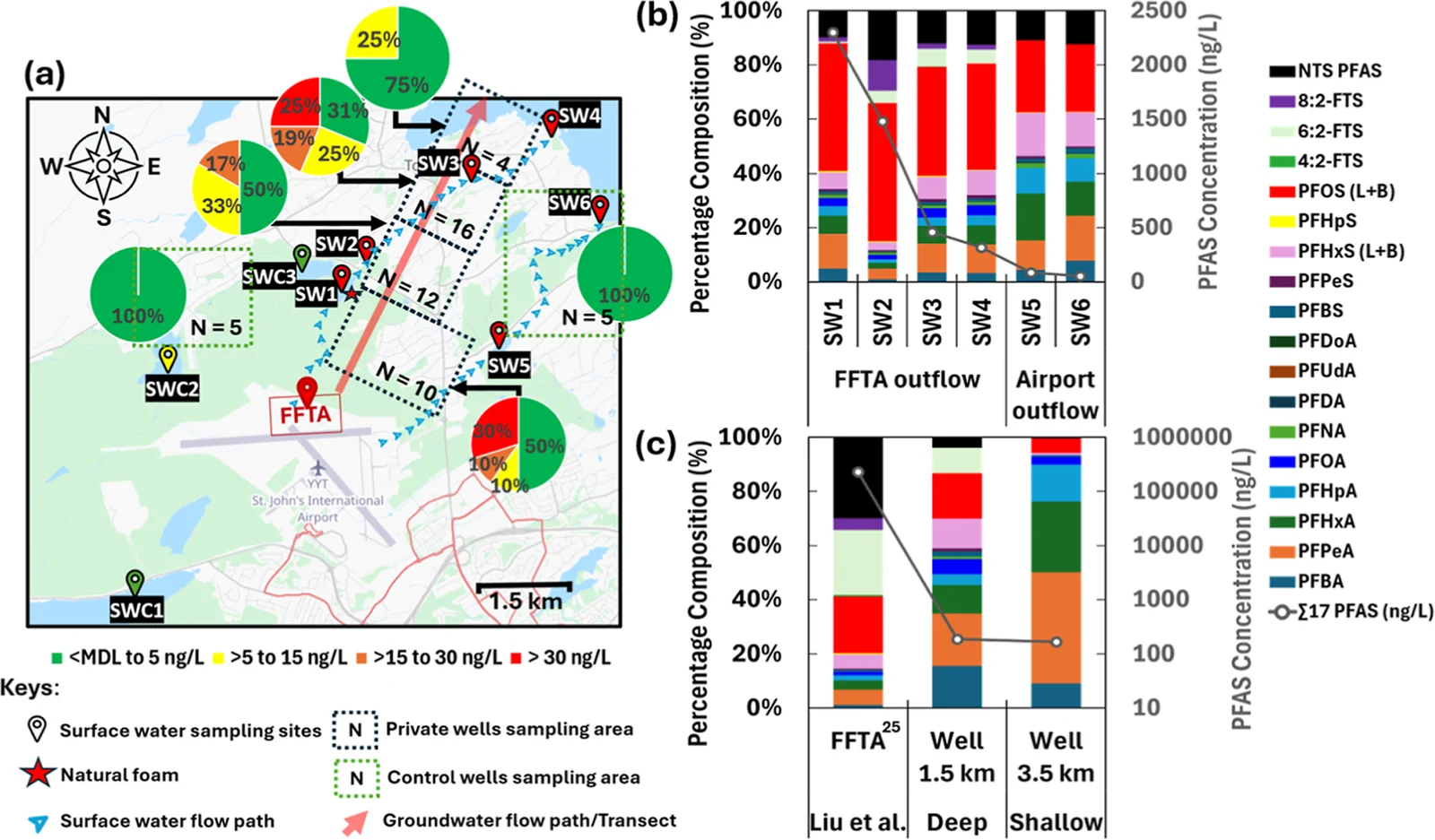
(a) Sampling locations and detection frequency of ∑17 targeted PFAS downgradient of the former Firefighter Training Area (FFTA) at St. John’s International Airport, NL, Canada, including surface water (SW, *n* = 6), natural foam (*n* = 1), and private wells (*n* = 42). Control surface water (SWC, *n* = 3) and wells (*n* = 10) are located outside the sampling transect. (b,c) Bar graphs showing the relative abundance of ∑17 targeted PFAS and ∑52 non-targeted PFAS (grey) in (b) surface water and (c) groundwater wells with exceedances >100 ng/L. Distance from the FFTA^25^ (km) is shown for each well. Map data © OpenStreetMap contributors.

Natural foam near the firefighter training facility (Figure 1a) was collected in a precleaned Ziploc bag, collapsed, and transferred to a 500 mL wide-mouth HDPE bottle, as described by Schwichtenberg et al.^43^ Collapsed foam samples were diluted 1:100 with methanol to obtain a 1 mL solution and spiked with internal and injection standards (10 μL each).

### 2.3 Instrumental Analysis

PFAS were analyzed using a Waters Cyclic IMS ion mobility mass spectrometer (Wilmslow, UK) coupled to a Waters Acquity H-class UPLC Ultrahigh performance liquid chromatograph (UHPLC) system by electrospray ionization and operated in the negative mode (ESI^–^). UHPLC separation employed a Raptor C18 column (100 mm × 2.1 mm × 1.8 μm). For targeted screening and NTS, 5 μL of sample extract was injected at 10 °C. The aqueous mobile phase (A) was 0.1% FA in LC–MS grade water, and the organic phase (B) was 0.1% FA in MeOH.^44,45^ Targeted screening did not employ cIMS separation, whereas NTS employed mobility-resolved data-independent acquisition (HDMS^E^). The MS was operated at 40,000 FWHM resolution across 50–1200 *m*/*z*. Full details of chromatographic conditions and cIMS-MS^2^ operating parameters are provided in the Supporting Information (Section S1).

### 2.4 Data Processing

#### 2.4.1 Non-targeted Screening (NTS) Workflow and Chemical Compound Identification

Suspect screening of previously identified PFAS was guided by libraries developed by Liu et al.^25^ and Joseph et al.^46^ Non-targeted screening (NTS) enabled the detection of PFAS not present in chemical structure databases. Both workflows were performed using the peak picking and data alignment procedures in Progenesis QI (Nonlinear Dynamics) applied to raw data files imported from Masslynx (Version 4.2). The software automatically selects a reference sample for ion map alignment (retention time and *m*/*z*), performs data compression, and deconvolutes ion signals into compound features using predefined adduct assignments ([M – H]^−^, [M – 2H]^2–^, and [2M – H]^−^).^47^ This yielded 39,680 unique features defined by mass-to-charge (*m*/*z*), retention time (RT, min), and collision cross-section (CCS, Å^2^). Subsequent processing was carried out in Microsoft Excel (described in Section 3.2), where blank-subtracted features were filtered to retain PFAS candidates using a CCS-to-m/z criterion adapted from MacNeil et al.^38^ (CCS <0.2 *m*/*z* + 90 Å^2^). Tentative assignments were made based on accurate mass (±5 ppm), isotope distribution ratios, and CCS (±2%) matching PFAS features in experimental libraries.^25,46^ In cases where reference CCS values were not available, a modeled CCS (±5%) (AllCCS2 ^48^) was predicted using a SMILES (Simplified Molecular Input Line Entry System) input. Confidence of feature assignments was reported using two complementary scales: Schymanski^49^ and Boatman scale,^50^ which incorporates mass accuracy, isotope distribution pattern, CCS agreement, and diagnostic fragment ions. Mobility resolved MS^2^ spectra were generated using the UNIFI software package (Waters Corporation).^51^ For clarity, confidence in the rest of the document will be annotated based on the Schymanski^49^ scale. Features with only elemental composition (within 5 ppm tolerance) but no structural matches were assigned a level 4 confidence, while compounds verified by authentic standards from the targeted analysis list were assigned level 1 confidence. The remaining compounds were broadly classified within level 2 and 3 confidences, depending on the MS^2^ fragmentation.

#### 2.4.2 Quantitative Analysis

Targeted PFAS (*n* = 23; Table S1) were quantified using isotopically labelled internal standards (Table S2). Calibration curves were constructed from authentic standards over an on-column range of 0.15–91 pg (5 μL injection), with internal standards (7.25–29 pg). Calibration curves were generated using 1/× weighted linear regression over the concentration range. All targeted compounds met acceptance criteria (*R* ^2^ > 0.990; Table S3) and were confirmed by retention time (±0.1 min) relative to their isotopically labelled analogue and by accurate mass (±5 ppm). Internal standards were also quantified using injection standards at concentrations of 7.25–21.75 pg. Method performance is summarized in Tables S2 and S3.

PFAS identified by NTS (Tables S4 and S5) were initially semi-quantified using a single-point calibration based on an injection standard (^13^C_4_–PFOS) to normalize instrument response, as presented in Figure 1b,c. These estimates were refined using a machine learning model (Quantem Analytics^52^) that predicts the instrument response factor from molecular structure (SMILES). Model performance was evaluated against calibration-derived results for 21 targeted PFAS in a subset of nine samples (*n* = 3 surface water, *n* = 6 groundwater), yielding a strong correlation (*R* ^2^ = 0.962; Figure S1a).

### 2.5 Quality Assurance and Quality Control (QA/QC)

All sampling and sample preparation procedures followed standard guidance for minimizing contamination.^53^ Fluoropolymer materials were avoided, nitrile gloves were changed frequently, and all containers and tubing were verified PFAS-free before use. Sampling bottles were pre-rinsed with ACS-grade methanol and LCMS-grade water. Field blanks (*n* = 8), collected alongside water samples, were subjected to identical handling and storage. Solvent blanks were analyzed to monitor potential laboratory contamination. Internal and injection standards were included in all samples and blanks to assess recovery. Average internal standard recoveries (*n* = 52) ranged from 60 to 121%, except for ^13^C_7_–PFUdA and ^13^C_1_–PFDoA, which exhibited lower recoveries (Table S2), consistent with WAX extraction efficiency for > C10 PFCAs.^54^ Natural foam samples were diluted 100-fold, and isotopically labelled internal standards were used to compensate for matrix effects (41–150%). Native spike recovery experiments (*n* = 3) are summarized in Table S3. Method detection limit (MDL) for each compound was calculated after blank subtraction as three times the standard deviation (3σ) of field blank concentrations (*n* = 8). MDLs for suspect and NTS features were estimated using peak intensity rather than concentration. Features were considered only when their intensity (after subtracting the average blank intensity) exceeded the estimated MDL (3σ). A minimum threshold of 1 was applied for features with MDLs below 1 to avoid background noise.

## 3 Results and Discussion

### 3.1 Spatial Distribution of Regulated PFAS in Surface Water and Groundwater

Seventeen of 23 targeted PFAS listed under the Canadian drinking water objective^16^ were detected in surface water samples (Table S1). In South Pond (SW1) and downstream sites (SW2–SW4), ∑17 PFAS concentration ranged from 318 to 2302 ng/L. Lower concentrations were observed at SW5 (85 ng/L), SW6 (52 ng/L), and control sites (<MDL–14.3 ng/L), shown in Figure 1a,b. This suggests that surface waters connected to the FFTA represent the primary PFAS transport pathway. Repeated sampling at SW1 and SW3 revealed temporal variability (RSD = 47–65%), highlighting the importance of multiple sampling events for characterizing PFAS exposure. PFOS (a major component of AFFFs) accounted for 62% of the ∑17 PFAS exposure near the source and decreased to 45% approximately 4.5 km downstream (Figure 1b). This decline is consistent with patterns previously observed from AFFF discharge sites in Toronto and Rhode Island.^4,55,56^ PFAS concentrations were also influenced by catchment boundaries, evidenced by SWC3, approximately 500 m from SW1, with no detectable PFAS (<MDL), despite its proximity to the FFTA.^41^ PFOS exceeded the highest concentration reported in Canadian surface waters over the past decade (150 ng/L) up to 3.5 km from the facility.^57–59^ Though concentrations remained well below the Canadian freshwater guideline of 6800 ng/L, they exceeded the non-lethal threshold for aquatic life (250 ng/L) in sites 1.5 km away.^16,60,61^

Thirteen of the 17 PFAS detected in surface waters were also detected in groundwater (32 of 42 wells), with concentrations ranging from 1–184 ng/L (Table S1). Transport Canada previously reported that about 28% of homes within 1.5 km of the source exceeded the Canadian drinking water objective (30 ng/L).^16,62^ In our study, exceedances were 30% at 1.5 km (Area 1) and 25% at 3.5 km (Area 3) (Figure 1a). The average well depth in the study area was 87 m, though six of the 42 sampled wells were classified as shallow (<30 m) within Areas 2 and 3.^41^ PFAS concentrations were not consistently influenced by well depth, as exceedances of 100 ng/L were observed in both shallow (<30 m) and deep (>30 m) wells, shown in Figure 1c. The low ∑17 PFAS concentrations (<30 ng/L) in Area 2 (Figure 1a) suggest an alternate transport mechanism to Area 3 wells, rather than direct groundwater transport from the FFTA. In the absence of other PFAS sources and given the proximity of Area 3 wells to SW3, a wetland along the FFTA flow path, contamination may reflect surface water–groundwater exchange. Tokranov et al.^33^ previously demonstrated that such exchange extended PFAS transport beyond 3 km from a contamination source, with a predominance of short-chain PFCAs (C_4_–C_7_). This pattern was consistent with observations from impacted wells near SW3, where short-chain PFCAs accounted for 63–95% of total PFCA concentrations. The variability in PFAS concentrations observed across multiple sampling events (RSD = 17–44%) further supports this interpretation, as surface water–groundwater mass transfer processes have previously been reported to cause fluctuations in PFAS concentrations.^33,63^

### 3.2 Identification of Unregulated PFAS in Surface Water and Groundwater

A total of 39,680 mass spectral features, characterized by *m*/*z*, RT, and CCS, were extracted from all surface water and groundwater samples. Of these, 5038 exceeded their associated MDL (see Section 2.5) and were most abundant in sites SW1–SW4 (Figure 1a) receiving outflow from the FFTA. Additional data filters were then applied to identify probable PFAS. A CCS-to-m/z filter adapted from MacNeil et al.^38^ was used to narrow the 5038 features to 261 (Figure 2a). Further reduction to 213 candidates was achieved by manually removing silicon-containing compounds with characteristic isotope ratios (M+1/M > 30%), poorly retained compounds (RT < 1 min), and ringing artefacts inherent to time-of-flight detectors.^64^ Recognizing that the mass spectrum of a perfluoroalkyl substance consists of multiple features, corresponding to isotopic peaks, fragments, adducts, and dimers, the prioritized list of features was manually grouped into 69 PFAS, sorted into 33 structural classes as depicted in Figure 2b. This number excludes isomers (e.g., linear vs. branched), and for some of the 69 PFAS, additional isomers could be resolved using ion mobility, listed in Tables S4 and S5, as described in Section 3.3. A limitation of the present workflow is the potential for false negatives from the CCS–*m*/*z* filter, which retains compounds containing >5 Fluorine atoms.^38^ Thus, ultrashort-chain PFAS, such as trifluoroacetic acid, may be excluded. This limitation should be considered when interpreting the apparent PFAS composition.

**Figure 2:**
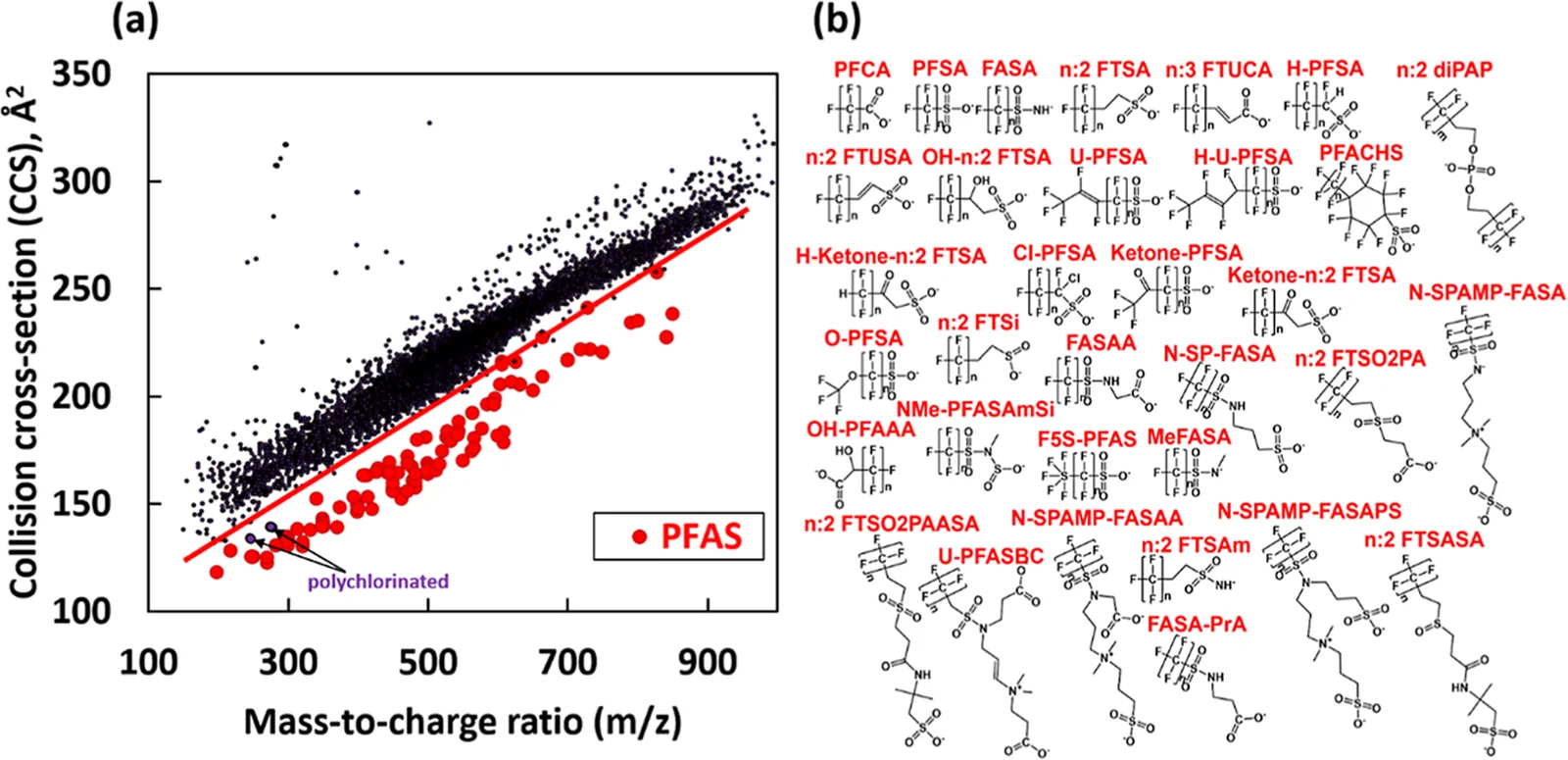
(a) m/z vs. CCS plot of the 5038 features detected in the surface and groundwater samples. The solid red line depicts the filter criterion proposed by MacNeil et al.^38^ The 69 PFAS are highlighted in red and the structures of the 33 classes of PFAS are shown in panel (b).

The PFAS detected included 17 regulated compounds whose identities were confirmed with authentic standards; 40 were tentatively identified by suspect screening, and 12 by NTS (Tables S4–S6). The confidence in these structure assignments was evaluated using both Schymanski and Boatman confidence scales (Tables S4 and S5). However, the assignments reported below are based on the Schymanski scale, as its Level 3 subclasses provide greater distinction among features with lower levels of structural confidence.^49,50^ PFAS whose *m*/*z*, RT, CCS and MS^2^ matched with authentic standards (*n* = 17) were assigned with the highest confidence (level 1), accounting for 25% of the total number of PFAS identified. The remaining 52 PFAS were classified as level 2 (*n* = 22, *m*/*z*, CCS [6 experimental; 16 predicted], and structure-diagnostic MS^2^ fragmentation), level 3a (*n* = 11, *m*/*z*, CCS [1 experimental; 10 predicted], non-specific MS^2^ fragmentation), level 3b (*n* = 16, *m*/*z*, CCS [4 experimental; 12 predicted]), and level 4 (*n* = 3, molecular formula, but unknown structure). Most of the PFAS identified were PFSAs, perfluoroalkane sulfonamides (FASAs), and fluorotelomer sulfonates (FTSs) and their derivatives (20%, 25%, and 26%, respectively). The evidence supporting their identification is further discussed in the sections below (3.2.1–3.2.3).

#### 3.2.1 Perfluoroalkanesulfonates (PFSAs) and PFSA Derivatives

The surface water samples (SW1-SW6) contained PFSAs (C_3_–C_10_) and perfluoroalkyl cyclohexanesulfonate (PFACHS, C_2_), which were used in AFFFs formulated between the 1970s and the early 2000s.^65,66^ Liu et al.^25^ also observed these chemicals in soil and groundwater of the FFTA, including C_2_–PFACHS. The C_4_–C_8_ PFSA homologues were confirmed by experiments with authentic standards (Figure S2), while the remaining homologues (C_3_, C_9_–C_10_) were supported by the observation of the structure-diagnostic *m*/*z* 79.9568 (SO_3_^–^) and *m*/*z* 179.9504 (C_2_F_4_SO_3_^–^) fragment ions, as well as RT and CCS values that are consistent with patterns extrapolated from the standards.

PFAS are known to travel long distances away from their source. For example, both the PFSAs (C_3_–C_8_) and C_2_–PFACHS were detected in groundwater and natural foams up to 3.5 km from the source of contamination (Figure 1a). Not all homologues behaved the same. For example, the natural foam concentrated long-chain homologues (C_8_–C_10_, Table S7). This is consistent with known partitioning behaviour of long-chain PFAS in freshwater foams.^43,67^ Table S7 summarizes the remaining 31 classes of PFAS and their detection in groundwater and natural foam.

PFOS and its homologues are widely regulated, whereas surface and groundwater samples also contained compounds closely similar to PFOS, but unregulated. These PFSA-derived chemicals include pentafluorosulfide PFSA (F5S-PFAS), hydrogen-substituted PFSA (H-PFSA), unsaturated PFSA (U-PFSA), hydrido unsaturated PFSA (H–U–PFSA), Ketone-PFSA, chlorine-substituted PFSA (Cl-PFSA), and oxa-PFSA (O-PFSA). They have been previously documented in AFFFs and groundwater at Canadian firefighter training facilities, including the upstream facility (MS^2^ spectra in Figures S3 and S4).^21,25,65^ Their presence raises the question about the true extent of PFAS exposure, addressed in Section 3.3.

#### 3.2.2 Perfluoroalkane Sulfonamides (FASAs) and FASA Precursors

Unregulated FASAs (C_3_–C_8_) were detected in surface water and groundwater wells, with C_6_ and C_8_ homologues enriched in natural foam. Identification was supported by the diagnostic fragment *m*/*z* 77.9650, SO_2_N^–^ (Figure S5), and the CCS values for their quasimolecular ions. FASAs are not listed in AFFFs, but older formulations (1970s–early 2000s) contained several compounds that can break down into FASAs.^21^ We detected a number of these FASA precursors in surface water, including *N*-SPAMP-FASAPS, *N*-SPAMP-FASAA, *N*-SPAMP-FASA, FASAA, and *N*-SP-FASA (Tables S6 and S7; Figures S6 and S7). Liu et al.^25^ also reported some of these compounds at the training facility.

Intermediate compounds formed during the breakdown of precursors, which eventually degrade into FASAs, were also present in surface and groundwater. These included MeFASA^22^ and previously undocumented products described below (Figure 3a,c,e). Perfluorohexane sulfonamido propionate (FHxSA-PrA) was detected at *m*/*z* 469.9732 (Figure 3a). The feature CCS (169.3 Å^2^; Figure 3a) closely matched the predicted CCS value for FHxSA-PrA (167.8 Å^2^; <2% difference), while differing from the reported experimental CCS value for the isomer *N*-methyl-perfluorohexane sulfonamido acetic acid (Me-FHxSAA, 176.1 Å^2^; >2% difference). The latter was not detected in the present study. The feature MS^2^ spectrum showed a neutral loss of C_3_H_4_O_2_, whereas Me-FHxSAA is expected to lose C_2_H_2_O_2_.^68^ FHxSA-PrA could potentially arise from dealkylation and oxidation of AFFF constituents, such as perfluorohexane amido betaine carboxylate (PFHxSBC), previously documented at the source.^25,69^ *N*-methyl-perfluorohexane sulfonamido sulfinate (NMe-PFHxSAmSi) and *N*-methyl-perfluorooctane sulfonamido sulfinate (NMe-PFOSAmSi) were also identified at *m*/*z* 475.9300 and 575.9235 (Figure 3c,e). These homologues produced diagnostic fragments at *m*/*z* 92.9884 and 156.9503, confirming an *N*-methyl sulfonamido-sulfinyl headgroup (SO_2_NCH_3_SO_2_^–^). Their CCS values aligned with predicted values, supporting the proposed structures. All three intermediates (FHxSA-PrA, NMe-PFOSAmSi, and NMe-PFHxSAmSi) have been observed in AFFF-exposed livestock, and our findings now demonstrate their presence in environmental waters, including branched NMe-PFOSAmSi isomers, observed for the first time.^70^

**Figure 3:**
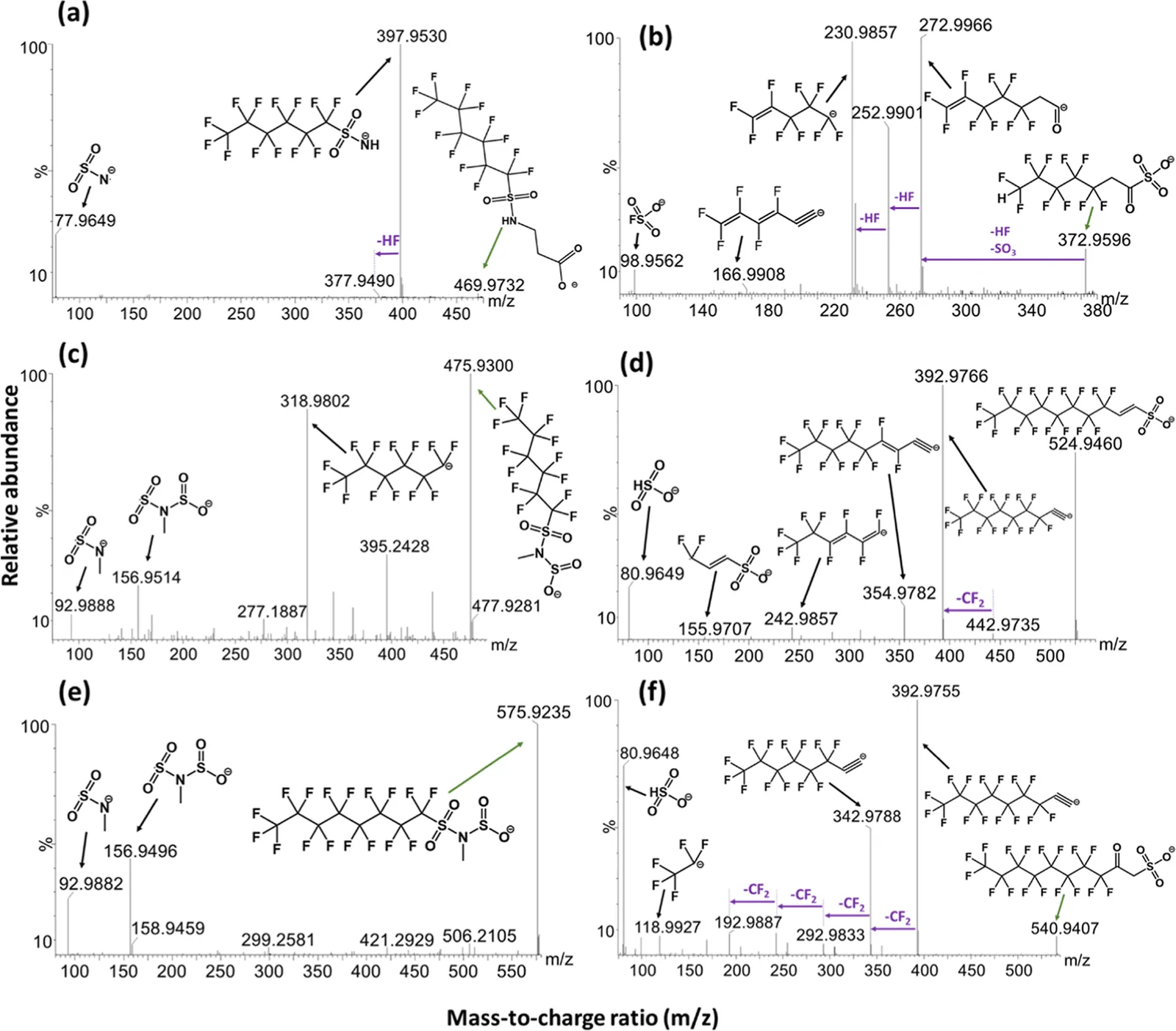
Annotated MS^2^ CID spectra of PFAS features characterized as (a) **FHxSA**-**PrA** (*m*/*z* 469.9732; CCS 169.3 Å^2^; *level 2*), (b) **nH**-**Ketone**-**5**:**2 FTS** (*m*/*z* 372.9596; CCS 148.5 Å^2^; *level 3a*), (c) **NMe**-**PFHxSAmSi** (*m*/*z* 475.9300; CCS 167.4 Å^2^; *level 2*), (d) **8**:**2 FTUS** (*m*/*z* 524.9460; CCS 181.2 Å^2^; *level 2*), (e) **NMe**-**PFOSAmSi** (*m*/*z* 575.9235; CCS 185.2 Å^2^; *level 2*), (f) **Ketone**-**8**:**2 FTS** (*m*/*z* 540.9407; CCS 182.5 Å^2^; *level 3a*). Details about their full names, mass errors and CCS errors are listed in Tables S3–S5. Collision energy (CE) = 50 ± 15 eV.

#### 3.2.3 Fluorotelomer Sulfonates (FTSs), FTS Precursors, and FTS Derivatives

FTSs (4:2, 6:2, and 8:2), the main components of modern AFFFs, were detected along with several precursors in surface water samples SW1–SW4 (Figure 1a; Table S7). Natural foam preferentially enriched 8:2 FTS, while only the 6:2 homologue was detected in groundwater and decreased with distance from the source. FTSs are unregulated but included in the recent Canadian drinking water objective and are under consideration for broader PFAS regulation.^60^ The FTSs (4:2, 6:2, and 8:2) were confirmed using authentic standards (Figure S8), while precursors (*n*:2 FTSASA, *n*:2 FTSO_2_PAASA, and *n*:2 FTSO_2_PA) were identified by diagnostic MS^2^ fragments (Figure S9) and CCS agreement (Tables S4 and S5). Liu et al.^25^ also observed these FTSs and precursors at the contamination source.

6:2- and 8:2 FTS undergo stepwise degradation to PFCAs, forming intermediate derivatives during the process that were also detected in the present study.^71,72^ Surface water and groundwater contained ketone-, hydroxy-, and unsaturated derivatives of 6:2 FTS (Ketone-6:2 FTS, OH-6:2 FTS, and 6:2 FTUS; Figures S10 and S11). Analogous 8:2 derivatives were also identified in surface water (8:2 FTUS, ketone-8:2 FTS, and OH-8:2 FTS). Most of these intermediates have been reported previously under controlled laboratory conditions and in AFFF-impacted waters, except 8:2 FTUS and ketone-8:2 FTS, which are observed for the first time.^72–74^ MS^2^ spectra (Figure 3d,f) showed diagnostic sulfonate (*m*/*z* 80.9646, HSO_3_^–^) and perfluoroalkyl (*m*/*z* 392.9760, C_9_F_15_^–^) fragments. This study also reports the first identification of an undocumented class of derivatives: hydrido-ketone-fluorotelomer sulfonates (nH-ketone FTS; Figure 3b and S11b). The MS^2^ spectrum of nH-ketone 5:2 FTS (Figure 3b) showed three sequential HF losses and diagnostic fragments at *m*/*z* 232.9837 (C_7_F_7_O^–^) and *m*/*z* 230.9856 (C_5_F_9_^–^), consistent with ketone substitution on a perfluoroalkyl chain. The experimental CCS (148.5 Å^2^) was smaller than all predicted positional isomers, matching only the 7H-positional isomer (153.8 Å^2^) within 3%. This likely reflects conformational folding driven by intramolecular hydrogen bonding between the polar headgroup and a residual hydrogen. Similar deviations between experimental and predicted CCS have been reported for hydrogen-substituted PFCAs, a behavior not captured by current prediction models (AllCCS2).^75^ As a result, the proposed structure is tentative (Schymanski level 3a) and the hydrogen and ketone substitution positions cannot be specified.

The tentatively identified nH-ketone FTSs (5:2 and 7:2) likely degraded from FTSs (6:2 and 8:2) via established pathways,^72^ followed by chain shortening^76^ and defluorination^77^ (Figure 4a). Chain shortening of FTSs typically occurs with a loss of sulfonate headgroup, but it has been observed to occur without headgroup removal in 6:2 FTS.^76^ Defluorination by hydrogen–fluorine (H/F) exchange has also been demonstrated in fluorotelomers at internal C–F bonds.^77^ However, the simultaneous occurrence of both chain shortening and defluorination within a single degradation pathway has not previously been documented. The proposed precursor-product relationship was supported by their co-occurrence: elevated 6:2 FTS corresponded with higher relative abundance of nH-ketone 5:2 FTS (Figure 4b), while a similar but weaker trend was observed for 8:2 FTS and nH-ketone 7:2 FTS. The lower abundance of nH-7:2 FTS likely reflects both low concentrations of 8:2 FTS (Figure 1) and the preferential microbial degradation of shorter-chain PFAS.^78^ Consistent with the proposed pathway in Figure 4a, PFAS distribution in groundwater shown in Figure 4c reveals that 6:2 FTS was most abundant near the source and declined downgradient, followed by the appearance of putative intermediate (OH-6:2 FTS, ketone-6:2 FTS and nH-ketone 5:2 FTS) and terminal (PFHpA) products.^72^ PFCAs can originate from multiple sources, but the detection of intermediate FTS degradation products may strengthen evidence of AFFF contamination in surface water and groundwater wells, which is further discussed in Section 3.4.

**Figure 4:**
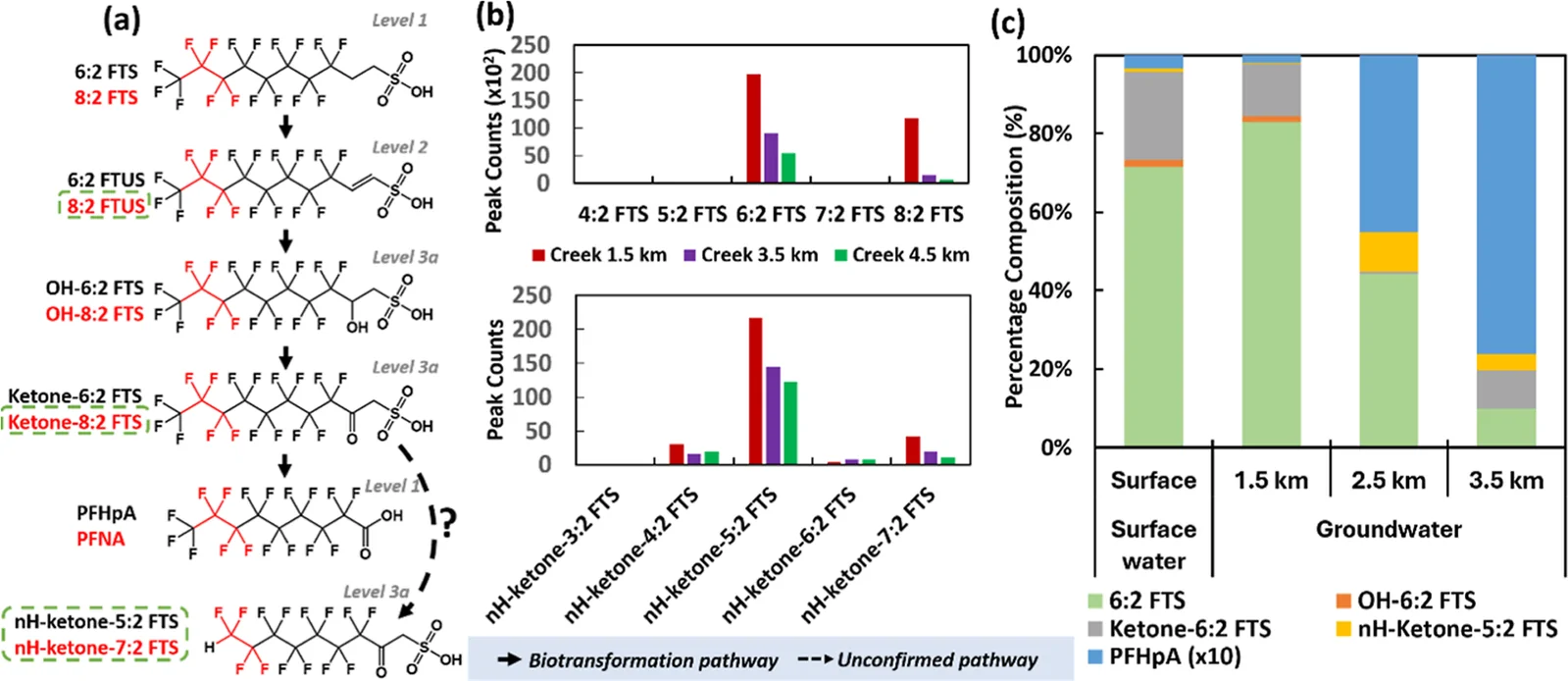
(a) Proposed degradation pathway of 6:2- and 8:2 FTS, showing detected degradation products and their corresponding confidence levels,^49,50^ with novel PFAS highlighted in ***green***. (b) Bar graphs showing abundance of FTSs and nH–ketone FTS in surface waters. (c) Average spatial distribution of 6:2 FTS derivatives in surface water (n_SW1–SW4_ = 4) and contaminated groundwater wells (n_1.5km_ = 3, n_3km_ = 2, and n_3.5km_ = 4) showing progressive biodegradation via the proposed pathway.

### 3.3 How Much do Unregulated PFAS Contribute to Exposure?

PFAS not currently included in the Canadian drinking water objective (52 of the 69 identified PFAS) were estimated to contribute 12–18% to total PFAS concentrations in surface water and <4% in groundwater (see black bars in Figure 1b,c). These unregulated PFAS were detected only in surface water and groundwater collected downgradient of the FFTA and were absent from control sites. Their lower contribution in groundwater than in surface water is consistent with downgradient attenuation and degradation into regulated terminal products (e.g. PFCAs).^27,33,55,79,80^ These estimates were obtained by assuming unregulated PFAS have the same response factor as the injection standard (^13^C_4_–PFOS). However, this semi-quantitative approach does not account for the fact that different compounds ionize to different extents. To address this limitation, the machine-learning model developed by Liigand et al.,^52^ was used to predict the ionization efficiencies of unregulated PFAS from their putative molecular structures. Validation against a set of nine samples (*n* = 3 surface water; *n* = 6 groundwater) showed that the predicted concentrations for the regulated PFAS were closely similar to those computed using internal calibration with authentic standards (*R* ^2^ = 0.962; Root mean square percentage error = 27%, see Figure S1a). In the same vein, when the machine-learning model was applied to unregulated PFAS, their estimated contributions to total PFAS in surface and groundwater only increased slightly to 17–24% and <6%, respectively (Figure S1b). The low proportion of unregulated PFAS implies that the Canadian drinking water objective adequately captures most of the exposure in drinking water impacted by AFFF. These estimates should be interpreted with caution, as validation included only 3 out of 33 PFAS classes detected by NTS. Hence, the reported *R* ^2^ and 27% error are not applicable across the full range of structurally diverse unregulated PFAS classes. This means greater uncertainty is expected for unregulated PFAS that were not represented during model development and validation.^52^

Natural foams in surface waters can act as concentrated sources of PFAS exposure for humans and wildlife.^81^ Foam collected near the firefighter training facility (Figure 1a) was orders of magnitude higher than in the underlying surface water, containing approximately 2.0 mg/L total PFAS, with PFOS accounting for 66% (1.32 mg/L).^82^ The PFOS concentration in foam was nearly double the highest freshwater foam concentration previously reported in Canada (0.772 mg/L)^83^ and consistent with prior studies that observed a similar enrichment.^43,67^ However, only 12/69 PFAS detected in surface waters were also concentrated in the foam (Table S7). For example, PFOS in AFFFs occurs as a mixture of linear (L) and branched (br) isomers,^84^ but as shown in Figure S12, only the linear (L-PFOS) and singly branched isomers (P1 – P6),^85^ were observed in foam, whereas the doubly branched PFOS isomers were not. The preferential enrichment of hydrophobic isomers (L-PFOS and P1–P6 isomers) in natural foam resembles patterns observed in wildlife, which suggests that natural foam may represent bioavailable PFAS in surface waters.^85,86^ In the present study, we identified two compounds (8:2 FTUS and Ketone-8:2 FTS) that have not been reported previously in natural foams.

### 3.4 Markers of PFOS and 6:2 FTS Contamination in Surface Water and Groundwater

Identifying the source of PFAS contamination typically relies on comparing isomer distributions and diagnostic ratios (such as PFOS/PFOA) in suspected source and receptor waters.^87–90^ However, these approaches may become less informative with increasing distance from the source as precursor compounds are depleted or diluted. In the present study, surface water samples (*n* = 6) showed PFOS/PFOA ratios ranging from 9 to 31:1. This is consistent with inputs from electrochemically fluorinated (ECF) legacy AFFFs, which contained high PFOS/PFOA ratios: Nailes et al.^88^ suggest a diagnostic ratio of >4:1, pure product formulations are typically characterized by a ratio of 90:1. In contrast, control sites exhibited PFOS/PFOA ratios of approximately 1–2:1. Equivalent diagnostic ratios are not established for FT-based AFFFs. Instead, their presence is inferred from the detection of 6:2 FTS. In the present study, the PFAS profiles were dominated by PFSAs with smaller contributions from FTSs, which is consistent with the use of both ECF-based and FT-based AFFF formulations at the FFTA.

Linking PFAS sources to groundwater contamination is challenging because subsurface transport alters PFAS profiles through sorption and degradation processes.^33,80^ For example, PFSAs sorb more strongly to organic matter and soil than their equivalent PFCAs, which skews PFSA/PFCA ratios with distance from the contamination source.^67,79^ PFOS isomer ratios are similarly affected, resulting in L/br ratios in groundwater that are <2:1.^89^ In the present study, L/br PFOS ratios were 3–5:1 in impacted surface waters, consistent with ECF inputs, but decreased to 1–3:1 in groundwater (p < 0.05; Figure S13).^89^ To strengthen the evidence linking AFFF contamination at the FFTA to receptor wells, we identified five compounds associated with PFOS and 6:2 FTS degradation pathways, shown in Figure 5. These compounds were detected in both contaminated surface waters and downgradient wells, with matching retention time (RT) and drift time (DT) supporting their identification in both media. These compounds are not specific to AFFF, and their presence alone cannot exclude contributions from other PFAS sources. However, given the connected flow path from the FFTA through the contaminated surface waters and downgradient wells, their occurrence in both media is consistent with transport from the FFTA to the receptor wells. Branched PFAS isomers are characteristic of ECF manufacturing processes.^89^ Markers associated with ECF inputs included branched PFOS (br-PFOS; Figure S12), H-PFOS, and NMe-PFOSAmSi.^65,76^ br-PFOS was detected in all 7 impacted wells (∑17 PFAS >30 ng/L), supporting ECF inputs at both 1.5 km and 3.5 km distances. H-PFOS is both a manufacturing byproduct and a PFOS biodegradation product. H-PFOS and NMe-PFOSAmSi were detected in 5 of 7 wells (*n*_1.5 km_ = 3/3; *n*_3.5 km_ = 2/4) and 4/7 wells (*n*_1.5 km_ = 2/3; *n*_3.5 km_ = 2/4), respectively. NMe-PFOSAmSi showed a relatively consistent distribution in surface water, contributing approximately 5–10% relative to H-PFOS along the established flow path. Similarly, in wells where it was detected, it accounted for 30% of their combined contribution. In contrast, its contribution in wells closer to the FFTA was more variable. Markers associated with 6:2 FTS included nH-ketone-5:2 FTS and ketone-6:2 FTS, representing different stages of 6:2 FTS degradation.^71,72^ Ketone-6:2 FTS was present in 4/7 wells, primarily in those closest to the source (*n*_1.5 km_ = 3/3; *n*_3.5 km_ = 1/4). Meanwhile, nH-ketone-5:2 FTS (reported here for the first time) was detected in 5/7 wells and increased in relative abundance over the intermediate portion of the groundwater flow path before declining further downgradient (Figures 4c and 5). Although its distribution is consistent with the proposed 6:2 FTS transformation pathway in Figure 4, this interpretation remains tentative in the absence of an authentic standard for confirmation. Nevertheless, its distribution suggests potential use as a long-range indicator of PFAS contamination in groundwater. Finally, PFECHS, a cyclic PFAS reported in certain AFFFs (1989) and airport hydraulic fluids, was detected in 6 out of 7 wells (*n*_1.5 km_ = 3/3; *n*_3.5 km_ = 3/4).^65,66^ Because PFECHS shares a similar *m*/*z* (460.9338) with U-PFOS, a PFOS degradation product, observed isomers may include contributions from both compounds. The most abundant isomer was confirmed as PFECHS based on its MS^2^ fragmentation (Figure S3a). The isomer distribution of PFECHS/U-PFOS in these wells (Figure 5) shows enrichment of branched (less hydrophobic) forms downgradient, similar to the behaviour observed in PFOS. Although no clear evidence of competing PFAS sources was identified, the presence of these compounds along the flow path connected to the FFTA suggested AFFF contribution, although their presence alone is not uniquely indicative of AFFFs.

**Figure 5:**
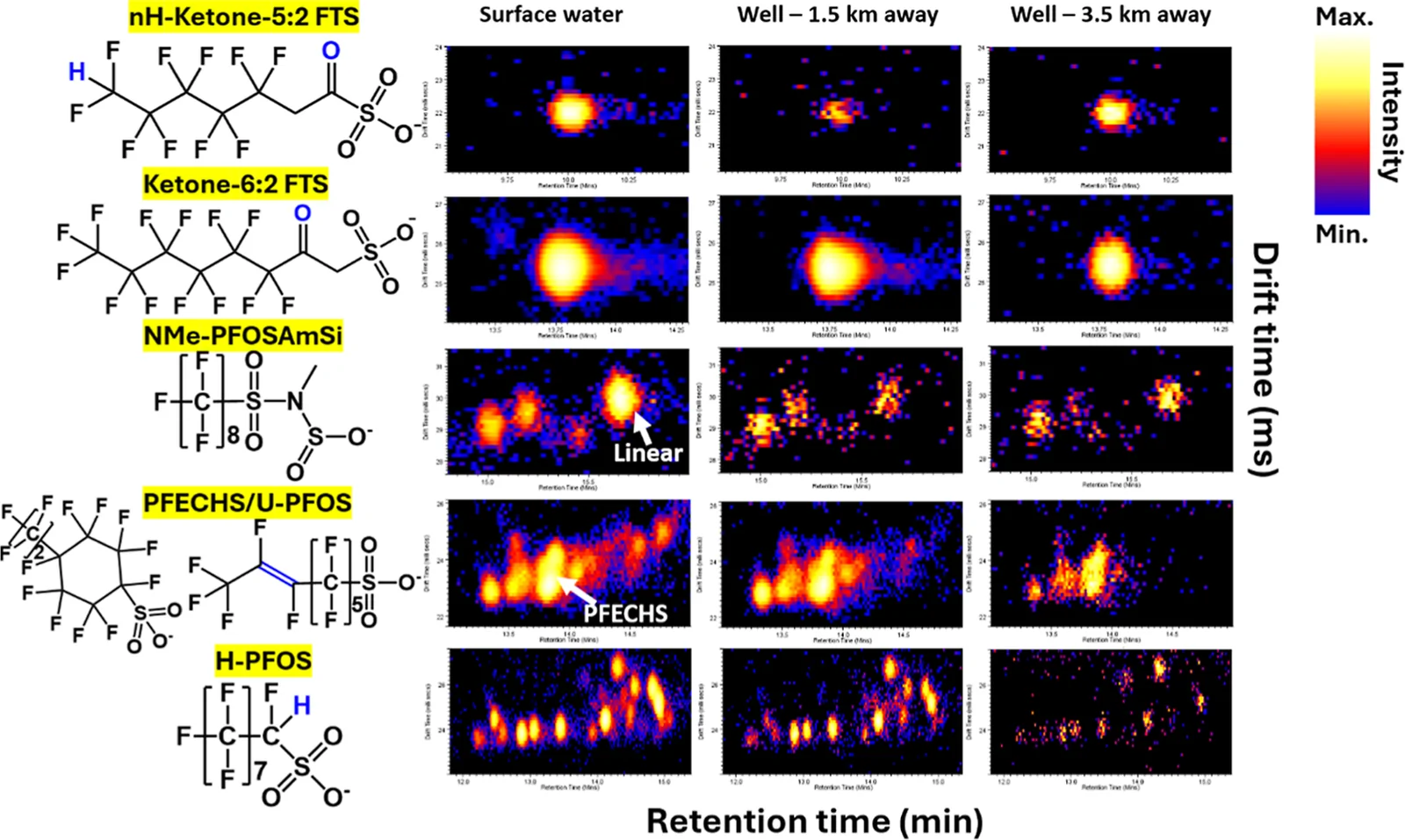
Heat maps showing extracted drift time vs. retention time of PFOS- and 6:2 FTS transformation products, detected across AFFF-impacted surface water and groundwater samples, including **nH**-**Ketone**-**5**:**2 FTS** (*m*/*z* = 372.9596; *level 3a*), **Ketone**-**6**:**2 FTS** (*m*/*z* = 440.9475; *level 3a*), **NMe**-**PFOSAmSi** (*m*/*z* = 575.9235; *level 2*), **PFECHS**/**U**-**PFOS** (*m*/*z* = 460.9338; *level 3a*), and **H**-**PFOS** (480.9401; level 3a). *Atoms and bonds in **blue** denote regions of positional isomerism.

### 3.5 Environmental Implications and Limitations

This study reveals PFAS migration from an FFTA at St. John’s International Airport into downstream surface waters and drinking wells. It also demonstrates the importance of NTS in characterizing unregulated PFAS transformation products in the environment. The results showed that routinely monitored PFAS prioritized under the Canadian drinking water objectives account for the majority of PFAS burden associated with AFFFs.^16^ The observations of the hotspot investigated in the present study are likely representative of other FFTA sites that have been inactive for >20 years.^11^ However, it is important to recognize that PFAS profiles at more recently active sites (or other sources of PFAS^32,91,92^ ) may vary due to changes in product formulations,^65,93^ and environmental transformation processes.^27,69,76,94^ The minor transformation products, including the six previously undocumented compounds identified in the present study, provided insight into PFAS transport and degradation. These transformation products may preserve information on PFAS degradation pathways and provide complementary evidence for source attribution in contaminated wells where precursors may be depleted.^72,76,94^ Spatial patterns demonstrated that PFAS distribution is governed by hydrological connectivity and catchment boundaries rather than distance from the contamination source alone. These findings highlight the limitations of monitoring strategies based solely on proximity to a hotspot. Additionally, variability in PFAS concentrations between sampling events indicates that single-time-point sampling may not adequately represent drinking-water exposure, particularly in wells influenced by surface water–groundwater exchange.^33^ Twenty-five to 30 percent of wells near the FFTA and a hydrologically connected wetland exceeded the Canadian drinking-water objective^16^ of 30 ng/L for ∑17 PFAS. The variability among wells in these impacted areas may reflect differences in aquifer properties.^95^ These findings highlight areas with elevated contamination risks, but due to the limited sample size, they do not reflect individual exposures. According to Health Canada,^16,96^ it is recommended that individuals within such areas perform routine PFAS screening of their wells and take adequate precautions to mitigate exposure.^16,97^

## Supplementary Material



## Data Availability

The authors confirm that the data supporting the findings of this study are available within the article and the Supporting Information.

## Notes

Additional experimental details, including information on analytical standards, method performance, and collision-induced dissociation mass spectra and collision cross sections of (non)­targeted compounds (PDF)
