# Supplementary material for: Identification of Novel PFAS Transformation Products in AFFF-Impacted Surface and Groundwater
Source: Environ Sci Technol. 2026 Oct 14;60(44):32110–22. doi: 10.1021/acs.est.6c11616 (PMC13757950; doi:10.1021/acs.est.6c11616)
Supplement: Supplementary file 1 [file es6c11616_si_001.pdf]

Supporting information

Identification of novel PFAS transformation products in AFFF-impacted surface and groundwater

Emmanuel C. Tolefe[1], Gavyn C. Lastoria[1], Jaanus Liigand[2], Lindsay S. Cahill[1], and Karl J. Jobst[1,*]

1 – *Department of Chemistry, Memorial University of Newfoundland, 45 Arctic Ave., St. John's, NL A1C 5S7, Canada*

2 – Quantem Analytics, 51008 Tartu, Estonia

*Corresponding author: kjobst@mun.ca

Supporting Information contains:

27 pages
7 tables
13 figures

Tables		Pages
S1	List of targeted per- and polyfluoroalkyl substances (n = 23), their full names, and detection frequencies (freq.) across 42 groundwater samples (GW), 6 surface water samples (SW), 10 control groundwater samples (GWC), and 3 control surface water samples (SWC).	5
S2	Summary of internal standard recoveries (%) and corresponding analytes across groundwater samples (n = 42) and controls (n = 10).	6
S3	Summary of LC-MS method performance in per-/polyfluoroalkyl substances (PFAS) quantitation.	7
S4	*Level 2* confidence identifications of suspect and non-target PFAS including their acronyms, theoretical and observed m/z, retention time (RT), number of isomers detected, predicted CCS (experimental indicated by ††), and feature CCS.	8
S5	*Level 3 & 4* confidence identifications of suspect and non-target PFAS including their acronyms, theoretical and observed m/z, retention time (RT), number of isomers detected, predicted CCS (experimental indicated by ††), and feature CCS.	9
S6	Full names and SMILES of per- and polyfluoroalkyl substances (PFAS) identified by target, suspect, and non-targeted screening	10-12

S7	List of PFAS classes and homologues detected in natural foam, surface waters (SW1–SW4; n = 4), and groundwater wells ($n_{1.5\ km}$ = 3; $n_{3.5\ km}$ = 4) with ∑17 PFAS > 30 ng/L, as well as documented detections at the former firefighter training facility (FFTA).	13

**Figures**		**Pages**

S1	Scatterplot comparing (a) the sum concentration of 21 targeted PFAS (exc. PFBA and PFPeA) estimated using a 5-point calibration curve versus a machine-learning model prediction; and (b) the sum concentration of 49 non-targeted PFAS (*Schymanski level 2, 3a, and 3b*) estimated using a single-point calibration ($^{13}C_4$-PFOS) and the machine-learning model in nine representative water samples, including 3 surface water and 6 groundwater samples.	14
S2	Annotated MS^2 CID spectra of (a) **PFOS** and (b) **PFOA**, comparing an authentic analytical standard (top) against a surface water sample collected from South Pond (bottom). Overlapping fragment ions illustrate structural similarity. Collision Energy (CE) = 35 ± 15V	15
S3	Annotated MS^2 CID spectra of unknown features (a) **PFECHS** (m/z 460.9338, *level 2*); and (b) **F5S-PFOS** (m/z 606.8945, *level 2*). Collision Energy (CE) = 35 ± 15V.	16
S4	Annotated high-energy spectra of unknown features (a) **PFPrS** (m/z 248.9461, *level 2*), (b) **H-PFOS** (m/z 480.9401, *level 3a*), (c) **Ketone-PFOS** (m/z 476.9283, *level 3a*), (d) **H-U-PFOS** (m/z 442.9439, *level 3a*). Collision energy (CE) = 20 – 50V.	17
S5	Annotated high-energy spectra of unknown features (a) **FPrSA** (m/z 247.9626, *level 2*), (b) **FBSA** (m/z 297.9591, *level 2*), (c) **FPeSA** (m/z 347.9562, *level 2*), (d) **FHxSA** (m/z 397.9528, *level 2*). Collision energy (CE) = 20 – 50V.	18
S6	Annotated MS^2 CID spectra of unknown features (a) **SPAMP-FHxSA** (m/z 605.0457, *level 2*), (b) **SPAMP-FHxSAA** (m/z 663.0510, *level 2*), (c) **SPAMP-FHxSAPS** (m/z 727.0495, *level 2*) (d) **SPAMP-FOSAPS** (m/z 827.0419, *level 2*). Collision energy (CE) = 35 ± 15V.	19
S7	Annotated MS^2 CID spectra of unknown features (a) **FHxSAA** (m/z 455.9585, *level 2*), (b) **N-SP-FHxSA** (m/z 519.9568, *level 2*). Collision energy (CE) = 35 ± 15V.	20
S8	Annotated MS^2 CID spectra of (a) **8:2 FTS** and (b) **6:2 FTS**, comparing an authentic analytical standard (top) against a surface water sample collected	21

	from South Pond (bottom). Overlapping fragment ions illustrate structural similarity. Collision Energy (CE) = 35 ± 15V.	
S9	Annotated MS^2 CID spectra of unknown features (a) **6:2 FTSASA** (m/z 602.0341, *level 2*), (b) **n:2 FTSO2PAASAs, n = 3** (m/z 618.0295, *level 2*), (c) **n:2 FTSO2PAASAs, n = 4** (m/z 718.0233, *level 2*). Collision energy (CE) = 35 ± 15V	22
S10	Annotated MS^2 CID spectra of unknown features (a) **6:2 FTUS** (m/z 424.9528, *level 2*), (b) **Ketone-6:2 FTS** (m/z 440.9475, *level 3a*), (c) **OH-8:2 FTS** (m/z 542.9561, *level 3a*), MS^2 CID Spectrum. Collision Energy (CE) = 35 ± 15V.	23
S11	Annotated MS^2 CID spectra of unknown features (a) **OH-6:2 FTS** (m/z 442.9630, *level 3a*), (b) **nH-Ketone-7:2 FTS** (m/z 472.9537, *level 3a*), (c) **6:2 diPAP** (m/z 788.9751, *level 2*). Collision energy (CE) = 35 ± 15V.	24
S12	Heatmap showing **RT** (min) vs. **Drift time** (ms) of perfluorooctane sulfonate (PFOS) isomers resolved under a 78 ms multipass cIMS separation, in South Pond (SW1), natural foam, and groundwater wells located 1.5 km and 3.5 km away from the firefighter training facility with ∑17 PFAS > 100 ng/L.	25
S13	Boxplots comparing the **linear-to-branched (L:br) PFOS isomer ratios** in surface water sites (SW1–SW6, n = 6) and groundwater wells (n = 25) between 1.5 km and 4.5 km from the former firefighter training area (FFTA), containing detectable concentrations of PFOS.	26

Section S1: Liquid Chromatograph and Mass Spectrometer Operating Conditions

Ultra-High Performance Liquid Chromatography (UHPLC) separation employed a Raptor C18 column (100 mm × 2.1 mm × 1.8 μm). For targeted screening, 5 μL of sample extract was injected at 10 °C. The aqueous mobile phase (A) was 0.1% Formic acid (FA) in LC-MS grade water, and the organic phase (B) was 0.1% FA in methanol (MeOH). FA was included in both mobile phases to improve chromatographic performance by enhancing peak shape and separation. Although acidic mobile-phase modifiers can suppress ionization in negative electrospray, isotopically labelled PFAS internal standards were used to compensate for matrix effects and variations in ionization efficiency.[1,2] Elution was performed at 0.3 mL/min, beginning at 95% A, ramping to 75% A in 0.5 min, then to 5% A at 7 min. This composition was held until 8.5 min, returned to initial conditions by 9 min, and maintained for a total runtime of 11 min at 25 °C. For non-targeted screening (NTS), the UHPLC flow rate was decreased to 0.2 mL/min, with the same mobile phases but an extended gradient. The initial composition was held for 4 min, ramped to 5% A by 17 min, maintained until 25 min, and returned to initial conditions for a total runtime of 30 min.

PFAS were analyzed using a Waters Cyclic IMS ion mobility mass spectrometer (Wilmslow, UK) coupled to a Waters Acquity H-class UPLC Ultrahigh performance liquid chromatograph (UHPLC) system by electrospray ionization and operated in the negative mode (ESI^-). The Mass Spectrometer (MS) was operated at 40,000 FWHM resolution, with a capillary voltage of 0.5 kV, source temperature of 100 °C, and desolvation temperature of 350 °C. MS acquisition was performed across 50–1200 m/z with a 1.0 s scan time during targeted screening. Post-run lockmass calibration was performed using $^{13}C_4$-PFOS ($^{13}C_4C_4SO_3^-$ at m/z 502.9434). While targeted screening did not employ ion mobility separation, non-targeted screening employed cIMS for orthogonal separation. cIMS separation employed N_2 drift gas at 3.5 L/min, and a cyclic ion mobility cell with a traveling wave height of 15 V, operated in single pass (2 ms separation) and multipass (78 ms separation) to resolve isomeric PFAS. Mobility resolved data-independent acquisition ($HDMS^E$) was performed over 50–1200 m/z, with a 0.5 s scan time and a ramped MS^2 collision energy of 20–50 eV. Collision cross-section (CCS) calibration[3] was performed using the Waters Major Mix standard (22 compounds) and a real-time calibration adjustment using leucine enkephalin ($C_{28}H_{37}N_5O_7^-$) at m/z 554.2615.

Table S1: List of targeted per- and polyfluoroalkyl substances (n = 23), their full names, and detection frequencies (freq.) across 42 groundwater samples (GW), 6 surface water samples (SW), 10 control groundwater samples (GWC), and 3 control surface water samples (SWC).

Compound	Full name	GW (freq.)	SW (freq.)	GWC (freq.)	SWC (freq.)
**PFBA**	Perfluorobutanoic acid	12/42	6/6	n.d.	2/3
**PFPeA**	Perfluoropentanoic acid	20/42	6/6	n.d.	1/3
**PFHxA**	Perfluorohexanoic acid	18/42	6/6	n.d.	1/3
**PFHpA**	Perfluoroheptanoic acid	12/42	6/6	n.d.	2/3
**PFOA**	Perfluorooctanoic acid	12/42	4/6	1/10	n.d.
**PFNA**	Perfluorononanoic acid	2/42	6/6	n.d.	n.d.
**PFDA**	Perfluorodecanoic acid	1/42	6/6	n.d.	n.d.
**PFUdA**	Perfluoroundecanoic acid	n.d.	5/6	n.d.	n.d.
**PFDoA**	Perfluorododecanoic acid	n.d.	1/6	n.d.	n.d.
**PFBS**	Perfluorobutane sulfonate	9/42	6/6	n.d.	n.d.
**PFPeS**	Perfluoropentane sulfonate	3/42	6/6	n.d.	n.d.
**PFHxS**	Perfluorohexane sulfonate	15/42	6/6	n.d.	1/3
**PFHpS**	Perfluoroheptane sulfonate	1/42	6/6	n.d.	n.d.
**PFOS**	Perfluorooctane sulfonate	25/42	6/6	n.d.	2/3
**4:2-FTS**	4:2 fluorotelomer sulfonate	n.d.	3/6	n.d.	n.d.
**6:2-FTS**	6:2 fluorotelomer sulfonate	3/42	4/6	n.d.	n.d.
**8:2-FTS**	8:2 fluorotelomer sulfonate	n.d.	4/6	n.d.	n.d.
**PFEESA**	Perfluoro(2-ethoxyethane) sulfonic acid	n.d.	n.d.	n.d.	n.d.
**NFDHA**	Nonafluoro-3,6-dioxaheptanoic acid	n.d.	n.d.	n.d.	n.d.
**GenX**	Hexafluoropropylene oxide dimer acid	n.d.	n.d.	n.d.	n.d.
**ADONA**	4,8-Dioxa-3H-perfluorononanoic acid	n.d.	n.d.	n.d.	n.d.
**9Cl-PF3ONS**	9-chlorohexadecafluoro-3-oxanonane-1-sulfonic acid	n.d.	n.d.	n.d.	n.d.
**11Cl-PF3OUdS**	11-chloroeicosafluoro-3-oxaundecane-1-sulfonic acid	n.d.	n.d.	n.d.	n.d.

*“**n.d.**”: Detection frequencies of zero (0).*

Table S2: Summary of internal standard recoveries (%) and corresponding analytes across groundwater samples (n = 42) and controls (n = 10).

Internal standard	Injection standard	Int. Std. recovery (%)	RSD (%)	Analyte quantified
**$^{13}C_5$-PFPeA**	$^{13}C_2$-PFOA	93	34	PFBA, PFPeA
**$^{13}C_5$-PFHxA**	$^{13}C_2$-PFOA	82	26	PFHxA
**$^{13}C_4$-PFHpA**	$^{13}C_2$-PFOA	87	25	PFHpA, GenX
**$^{13}C_8$-PFOA**	$^{13}C_2$-PFOA	86	24	PFOA
**$^{13}C_9$-PFNA**	$^{13}C_2$-PFOA	87	38	PFNA
**$^{13}C_6$-PFDA**	$^{13}C_2$-PFOA	60	25	PFDA
**$^{13}C_7$-PFUdA**	$^{13}C_2$-PFOA	40	20	PFUdA
**$^{13}C_1$-PFDoA**	$^{13}C_2$-PFOA	15	9	PFDoA
**$^{13}C_3$-PFBS**	$^{13}C_4$-PFOS	87	31	PFBS
**$^{13}C_3$-PFHxS**	$^{13}C_4$-PFOS	86	30	PFPeS, PFHxS
**$^{13}C_8$-PFOS**	$^{13}C_4$-PFOS	81	35	PFHpS, PFOS, PFEESA, ADONA, 9Cl-PF3ONS, 11Cl-PF3OUdS
**$^{13}C_2$-4:2 FTS**	$^{13}C_4$-PFOS	121	46	4:2 FTS, NFDHA
**$^{13}C_2$-6:2-FTS**	$^{13}C_4$-PFOS	116	64	6:2 FTS
**$^{13}C_2$-8:2-FTS**	$^{13}C_4$-PFOS	104	63	8:2 FTS

Table S3: Summary of LC-MS method performance in per-/polyfluoroalkyl substances (PFAS) quantitation.

Compound	RT (min)	MDL (ng/L)	Avg. native recovery (%)	RSD (%)	Linear range (pg)	Linearity
**PFBA**	3.77	2.8	89	5	0.15 - 91	0.9918
**PFPeA**	5.46	0.2	103	1	0.15 - 91	0.9966
**PFHxA**	6.54	0.09	118	2	0.15 - 91	0.9954
**PFHpA**	7.31	0.6	124	6	0.15 - 91	0.9963
**PFOA**	7.95	2.4	130	6	0.15 - 91	0.9971
**PFNA**	8.50	0.05	119	3	0.15 - 91	0.9952
**PFDA**	8.97	0.03	127	6	0.15 - 91	0.9951
**PFUdA**	9.44	0.01	127	4	0.15 - 91	0.9948
**PFDoA**	9.94	0.05	72	4	0.15 - 18	0.9997
**PFBS**	5.68	0.01	121	4	0.15 - 91	0.9971
**PFPeS**	6.59	0.01	119	13	0.15 - 91	0.9926
**PFHxS**	7.27	0.01	125	5	0.15 - 91	0.9959
**PFHpS**	7.88	0.01	119	3	0.15 - 91	0.9983
**PFOS**	8.41	0.02	126	7	0.15 - 91	0.9979
**4:2-FTS**	6.35	0.2	111	3	0.15 - 91	0.9984
**6:2-FTS**	7.83	0.1	110	9	0.15 - 91	0.9955
**8:2-FTS**	8.90	0.01	121	6	0.15 - 91	0.9982
**PFEESA**	6.08	0.01	130	23	0.15 - 91	0.9988
**NFDHA**	6.35	0.8	142	18	0.15 - 91	0.9924
**GenX**	6.77	0.9	118	18	0.15 - 91	0.9988
**ADONA**	7.34	0.1	179	5	0.15 - 91	0.9911
**9Cl-PF3ONS**	8.65	0.01	110	20	0.15 - 91	0.9986
**11Cl-PF3OUdS**	9.49	0.1	60	45	0.15 - 91	0.9927

Method detection limit (MDL) estimated using (n = 8) field blanks
Native recovery quantified in (n = 3) native-spiked samples

Table S4: *Level 2* confidence identifications of suspect and non-target PFAS including their acronyms, theoretical and observed m/z, retention time (RT), number of isomers detected, predicted[4] CCS (experimental[5] indicated by [††]), and feature CCS.

Proposed compound	Conf level		Molecular formula	Feature m/z	Retention time (min)	# Isomers	Adduct	Feature CCS (Å^2)	Predicted CCS (Å^2)	ΔCCS (%)	Theoretical m/z	Δm/z (ppm)
FPrSA	2	2*	$C_3H_2F_7NO_2S$	247.9626	8.78	1	M-H	125.5	128.9	2.6	247.9616	3.9
PFPrS	2	2*	$C_3HF_7O_3S$	248.9461	6.91	1	M-H	125.5	125.1[††]	0.3	248.9457	1.7
FBSA	2	2*	$C_4H_2F_9NO_2S$	297.9591	10.80	2	M-H	134.6	135.0[††]	0.3	297.9584	2.5
FPeSA	2	2*	$C_5H_2F_{11}NO_2S$	347.9562	12.57	2	M-H	143.4	145.6	1.5	347.9553	2.6
FHxSA	2	2*	$C_6H_2F_{13}NO_2S$	397.9528	14.06	3	M-H	149.9	152.0[††]	1.3	397.9521	1.9
6:2 FTUS	2	2*	$C_8H_3F_{13}O_3S$	424.9528	14.15	1	M-H	165.0	167.7	1.6	424.9517	2.5
FHxSAA	2	2*	$C_8H_4F_{13}NO_4S$	455.9585	14.88	1	M-H	164.6	165.1[††]	0.3	455.9575	2.1
PFECHS	2	2*	$C_8HF_{15}O_3S$	460.9338	13.97	6	M-H	154.5	153.3[††]	0.8	460.9329	1.9
FHxSA-PrA	2	2*	$C_9H_6F_{13}NO_4S$	469.9732	15.06	3	M-H	169.3	167.8	0.9	469.9732	0.0
PFHxSAmSi	2	2*	$C_7H_4F_{13}NO_4S_2$	475.9300	12.76	2	M-H	167.4	170.8	2.0	475.9296	1.1
N-SP-FHxSA	2	2*	$C_9H_8F_{13}NO_5S_2$	519.9568	14.23	2	M-H	182.9	178.1	2.7	519.9558	1.9
8:2 FTUS	2	2*	$C_{10}H_3F_{17}O_3S$	524.9460	17.04	1	M-H	181.2	183.7	1.4	524.9453	1.3
PFOSAmSi	2	2*	$C_9H_4F_{17}NO_4S_2$	575.9235	15.73	3	M-H	185.2	187.7	1.3	575.9232	0.7
6:2 FTSASA	2	2*	$C_{15}H_{18}F_{13}O_5NS_2$	602.0341	14.04	1	M-H	206.0	200.4	2.8	602.0341	0.0
SPAMP-FHxSA	2	2*	$C_{14}H_{20}F_{13}N_2O_5S_2^+$	605.0457	13.11	2	M^+-2H	216.5	204.4	5.9	605.0450	1.2
F5S-PFOS	2	2*	$C_8HF_{21}O_3S_2$	606.8945	18.08	2	M-H	183.5	191.3	4.1	606.8953	1.3
n:2 FTSO2PAASAs n=3	2	2*	$C_{15}H_{18}F_{13}NO_6S_2$	618.0295	14.61	1	M-H	207.3	203.7	1.7	618.0290	0.8
SPAMP-FHxSAA	2	2*	$C_{16}H_{22}F_{13}N_2O_7S_2^+$	663.0510	12.90	1	M^+-2H	227.5	218.2	4.2	663.0505	0.8
n:2 FTSO2PAASAs n=4	2	2*	$C_{17}H_{18}F_{17}NO_6S_2$	718.0233	17.33	1	M-H	222.1	220.2	0.9	718.0226	1.0
SPAMP-FHxSAPS	2	2*	$C_{17}H_{26}F_{13}N_2O_8S_3^+$	727.0495	12.76	1	M^+-2H	241.5	228.2	5.8	727.0487	1.1
6:2 diPAP	2	2*	$C_{16}H_9F_{26}O_4P$	788.9751	20.94	1	M-H	234.6	233.9[††]	0.3	788.9745	0.8
SPAMP-FOSAPS	2	2*	$C_{19}H_{26}F_{17}N_2O_8S_3^+$	827.0419	15.80	1	M+-2H	258.0	245.5	5.1	827.0424	0.6

*Confidence levels are annotated using Schymanski[6] scale and Boatman[7] scale (indicated by *)*

Table S5: *Level 3 & 4* confidence identifications of suspect and non-target PFAS including their acronyms, theoretical and observed m/z, retention time (RT), number of isomers detected, predicted[4] CCS (experimental[5] indicated by ††), and feature CCS.

Proposed compound	Conf level		Molecular formula	Feature m/z	Retention time (min)	# Isomers	Adduct	Feature CCS (Å)	Predicted CCS (Å2)	ΔCCS (%)	Theoretical m/z	Δm/z (ppm)
5:3 FTUCA	3b	4*	$C_8H_3F_{11}O_2$	338.9882	15.29	1	M-H	152.6	149.6	2.0	338.9879	1.0
nH-Ketone-5:2 FTS	3a	3*	$C_7H_4F_{10}O_4S$	372.9596	10.23	1	M-H	148.5	153.8	3.5	372.9592	1.0
OH-PFHxAA	3b	4*	$C_8H_3F_{13}O_3$	392.9797	13.42	1	M-H	153.5	154.7	0.8	392.9796	0.2
6:2 FTSAm	3b	4*	$C_8H_6F_{13}NO_2S$	405.9778	14.25	1	M-H-HF	163.5	161.9	1.0	405.9771	1.7
6:2 FTSi	3b	4*	$C_8H_5F_{13}O_2S$	410.9730	14.94	1	M-H	163.5	166.2	1.6	410.9724	1.4
MeFHxSA	3b	4*	$C_7H_4F_{13}NO_2S$	411.9676	15.21	1	M-H	153.3	152.9[††]	0.2	411.9677	0.3
Ketone-6:2 FTS	3a	3*	$C_8H_3F_{13}O_4S$	440.9475	13.88	1	M-H	164.8	163.9	0.5	440.9466	2.1
H-U-PFOS	3a	3*	$C_8H_2F_{14}O_3S$	442.9439	15.12	1	M-H	169.6	163.5	3.7	442.9423	3.6
OH-6:2 FTS	3a	3*	$C_8H_5F_{13}O_4S$	442.9630	12.99	1	M-H	164.7	163.9	0.5	442.9623	1.7
FHpSA	3a	3*	$C_7H_2F_{15}NO_2S$	447.9497	15.36	2	M-H	159.7	161.1	0.9	447.9489	1.9
nH-Ketone-7:2 FTS	3a	3*	$C_9H_4F_{14}O_4S$	472.9541	13.74	1	M-H	-	168.5	-	472.9529	2.5
Ketone-PFOS	3a	3*	$C_8HF_{15}O_4S$	476.9283	14.50	1	M-H	161.1	168.7	4.5	476.9278	1.1
H-PFOS	3a	3*	$C_8H_2F_{16}O_3S$	480.9401	14.27	13	M-H	161.1	167.3	3.7	480.9391	2.1
n:2 FTSO2PAs n=3	3b	4*	$C_{11}H_9F_{13}O_4S$	482.9946	14.42	1	M-H	180.0	178.6	0.8	482.9936	2.2
FOSA	3a	3*	$C_8H_2F_{17}NO_2S$	497.9465	16.48	3	M-H	169.0	169.7[††]	0.4	497.9457	1.6
UPFNS	3b	4*	$C_9HF_{17}O_3S$	510.9310	15.40	1	M-H	165.7	172.8	4.1	510.9297	2.5
Cl-PFOS	3b	4*	$C_8HClF_{16}O_3S$	514.9016	15.94	1	M-H	170.5	173.3[††]	1.7	514.9002	2.7
O-PFOS	3b	4*	$C_8HF_{17}O_4S$	514.9235	16.10	1	M-H	168.9	173.1	2.4	514.9246	2.2
Ketone-8:2 FTS	3a	3*	$C_{10}H_3F_{17}O_4S$	540.9407	16.86	1	M-H	182.5	181.0	0.8	540.9402	1.0
H-U-PFDS	3b	4*	$C_{10}H_2F_{18}O_3S$	542.9384	17.90	1	M-H	188.4	177.7	6.0	542.9359	4.6
OH-8:2 FTS	3a	3*	$C_{10}H_5F_{17}O_4S$	542.9561	16.14	1	M-H	184.0	180.2	2.1	542.9559	0.4
PFNS	3b	4*	$C_9HF_{19}O_3S$	548.9272	16.94	3	M-H	176.4	177.2[††]	0.5	548.9265	1.3
UPFDS	3b	4*	$C_{10}HF_{19}O_3S$	560.9267	16.67	1	M-H	174.8	180.8	3.3	560.9265	0.4
O-PFNS	3b	4*	$C_9HF_{19}O_4S$	564.9218	17.12	2	M-H	180.8	181.4	0.3	564.9214	0.6
8:2 FTSO2PA (n=4)	3b	4*	$C_{13}H_9F_{17}O_4S$	582.9881	16.77	1	M-H	196.6	194.7	1.0	582.9872	1.5
PFDS	3b	4*	$C_{10}HF_{21}O_3S$	598.9242	17.56	1	M-H	182.1	186.2[††]	2.2	598.9233	1.4
U-PFHxSBC	3b	4*	$C_{17}H_{19}F_{13}N_2O_6S$	625.0681	13.27	1	M-H	216.4	210.2	2.9	625.0678	0.5
UK-1	4	4*	$C_{10}H_9F_{13}N_2O_3S$	483.0052	14.63	1	M-H	177.0	-	-	483.0048	0.8
UK-2	4	4*	$C_{12}H_{13}F_{13}N_2O_3S$	511.0374	15.31	1	M-H	188.7	-	-	511.0361	2.5
UK-3	4	4*	$C_{14}H_{14}F_{16}N_2O_3S$	593.0421	13.46	1	M-H	206.1	-	-	593.0391	5.0

*Confidence levels are annotated using the Schymanski[6] scale and Boatman[7] scale (indicated by *)*

Table S6: Full names and SMILES of per- and polyfluoroalkyl substances (PFAS) identified by target, suspect, and non-target screening.

Compound	Full name	SMILES
PFBA	Perfluorobutanoic acid	C(=O)(C(C(C(F)(F)F)(F)F)(F)F)O
PFPeA	Perfluoropentanoic acid	C(=O)(C(C(C(C(F)(F)F)(F)F)(F)F)(F)F)O
PFHxA	Perfluorohexanoic acid	C(=O)(C(C(C(C(C(F)(F)F)(F)F)(F)F)(F)F)(F)F)O
PFHpA	Perfluoroheptanoic acid	C(=O)(C(C(C(C(C(C(F)(F)F)(F)F)(F)F)(F)F)(F)F)(F)F)O
PFOA	Perfluorooctanoic acid	C(=O)(C(C(C(C(C(C(C(F)(F)F)(F)F)(F)F)(F)F)(F)F)(F)F)(F)F)O
PFNA	Perfluorononanoic acid	C(=O)(C(C(C(C(C(C(C(C(F)(F)F)(F)F)(F)F)(F)F)(F)F)(F)F)(F)F)(F)F)O
PFDA	Perfluorodecanoic acid	C(=O)(C(C(C(C(C(C(C(C(C(F)(F)F)(F)F)(F)F)(F)F)(F)F)(F)F)(F)F)(F)F)(F)F)O
PFUdA	Perfluoroundecanoic acid	C(=O)(C(C(C(C(C(C(C(C(C(C(F)(F)F)(F)F)(F)F)(F)F)(F)F)(F)F)(F)F)(F)F)(F)F)(F)F)O
PFDoA	Perfluorododecanoic acid	C(=O)(C(C(C(C(C(C(C(C(C(C(C(F)(F)F)(F)F)(F)F)(F)F)(F)F)(F)F)(F)F)(F)F)(F)F)(F)F)(F)F)O
PFPrS	Perfluoropropane sulfonate	C(C(F)(F)F)(C(F)(F)S(=O)(=O)O)(F)F
PFBS	Perfluorobutane sulfonate	C(C(C(F)(F)S(=O)(=O)O)(F)F)(C(F)(F)F)(F)F
PFPeS	Perfluoropentane sulfonate	C(C(C(F)(F)F)(F)F)(C(C(F)(F)S(=O)(=O)O)(F)F)(F)F
PFHxS	Perfluorohexane sulfonate	C(C(C(C(F)(F)S(=O)(=O)O)(F)F)(F)F)(C(C(F)(F)F)(F)F)(F)F
PFHpS	Perfluoroheptane sulfonate	C(C(C(C(F)(F)F)(F)F)(F)F)(C(C(C(F)(F)S(=O)(=O)O)(F)F)(F)F)(F)F
PFOS	Perfluorooctane sulfonate	C(C(C(C(C(F)(F)S(=O)(=O)O)(F)F)(F)F)(F)F)(C(C(C(F)(F)F)(F)F)(F)F)(F)F
PFNS	Perfluorononane sulfonate	C(C(C(C(C(F)(F)F)(F)F)(F)F)(F)F)(C(C(C(C(F)(F)S(=O)(=O)O)(F)F)(F)F)(F)F)(F)F
PFDS	Perfluorodecane sulfonate	C(C(C(C(C(C(F)(F)S(=O)(=O)O)(F)F)(F)F)(F)F)(F)F)(C(C(C(C(F)(F)F)(F)F)(F)F)(F)F)(F)F
PFECHS	Perfluoro-4-ethylcyclohexane	C1(C(C(C(C(C1(F)F)(F)F)(F)S(=O)(=O)O)(F)F)(F)F)(C(C(F)(F)F)(F)F)F
H-PFOS	Hydrido-perfluorooctane sulfonate	C(C(C(C(C(C(C(F)(F)S(=O)(=O)O)(F)F)(F)F)(F)F)(F)F)(F)F)(C(F)(F)F)F
Ketone-PFOS	Keto-perfluorooctane sulfonate	C(=O)(C(C(C(C(C(C(F)(F)S(=O)(=O)O)(F)F)(F)F)(F)F)(F)F)(F)F)C(F)(F)F
F5S-PFOS	(n-Pentafluoro(5)sulfide)-perfluorooctane sulfonate	C(C(C(C(C(F)(F)S(F)(F)(F)(F)F)(F)F)(F)F)(F)F)(C(C(C(F)(F)S(=O)(=O)O)(F)F)(F)F)(F)F
Cl-PFOS	Chloro(Cl)- perfluorooctanesulfonate	C(C(C(C(C(F)(S(=O)(=O)O)Cl)(F)F)(F)F)(F)F)(C(C(C(F)(F)F)(F)F)(F)F)(F)F
O-PFOS	Perfluorooctane ether sulfonate	C(C(C(C(OC(F)(F)F)(F)F)(F)F)(F)F)(C(C(C(F)(F)S(=O)(=O)O)(F)F)(F)F)(F)F
O-PFNS	Perfluorononane ether sulfonate	C(C(C(C(C(F)(F)S(=O)(=O)O)(F)F)(F)F)(F)F)(C(C(C(OC(F)(F)F)(F)F)(F)F)(F)F)(F)F

H-U-PFOS	Hydrido-unsaturated perfluorooctane sulfonate	C(=C(/C(C(C(C(C(C(F)(F)F)(F)F)(F)F)(F)F)(F)F)(F)F)\F)\S(=O)(=O)O
H-U-PFDS	Hydrido-unsaturated perfluorodecane sulfonate	FC(/C(F)=C(F)/C(F)(F)F)([H])C(F)(F)C(F)(F)C(F)(F)C(F)(F)C(F)(F)C(F)(F)S(=O)(O)=O
UPFNS	Unsaturated perfluorononane sulfonate	C(=C(/C(F)(F)F)\F)(\C(C(C(C(C(C(F)(F)S(=O)(=O)O)(F)F)(F)F)(F)F)(F)F)(F)F)/F
UPFDS	Unsaturated perfluorodecane sulfonate	C(=C(/C(F)(F)F)\F)(\C(C(C(C(C(C(C(F)(F)S(=O)(=O)O)(F)F)(F)F)(F)F)(F)F)(F)F)(F)F)/F
FPrSA	perfluoropropane sulfonamide	C(C(F)(F)F)(C(F)(F)S(=O)(=O)N)(F)F
FBSA	perfluorobutane sulfonamide	C(C(C(F)(F)S(=O)(=O)N)(F)F)(C(F)(F)F)(F)F
FPeSA	perfluoropentane sulfonamide	C(C(C(F)(F)F)(F)F)(C(C(F)(F)S(=O)(=O)N)(F)F)(F)F
FHxSA	perfluorohexane sulfonamide	C(C(C(C(F)(F)S(=O)(=O)N)(F)F)(F)F)(C(C(F)(F)F)(F)F)(F)F
FHpSA	perfluoroheptane sulfonamide	C(C(C(C(F)(F)F)(F)F)(F)F)(C(C(C(F)(F)S(=O)(=O)N)(F)F)(F)F)(F)F
FOSA	perfluorooctane sulfonamide	C(C(C(C(C(F)(F)S(=O)(=O)N)(F)F)(F)F)(F)F)(C(C(C(F)(F)F)(F)F)(F)F)(F)F
SPAMP-FHxSAA	N-sulfopropyldimethylammoniopropyl-perfluorohexane sulfonamido acetic acid	C[N+](C)(CCCN(CC(=O)O)S(=O)(=O)C(C(C(C(C(C(F)(F)F)(F)F)(F)F)(F)F)(F)F)(F)F)CCCS(=O)(=O)[O-]
SPAMP-FHxSA	N-sulfopropyldimethylammonio propyl perfluoroalkanesulfonamide	C[N+](C)(CCCNS(=O)(=O)C(C(C(C(C(C(F)(F)F)(F)F)(F)F)(F)F)(F)F)(F)F)CCCS(=O)(=O)[O-]
SPAMP-FHxSAPS	perfluorohexane sulfonamido betaine disulfonate	C[N+](C)(CCCN(CCCS(=O)(=O)O)S(=O)(=O)C(C(C(C(C(C(F)(F)F)(F)F)(F)F)(F)F)(F)F)(F)F)CCCS(=O)(=O)[O-]
SPAMP-FOSAPS	perfluorooctane sulfonamido betaine disulfonate	C[N+](C)(CCCN(CCCS([O])(=O)=O)S(=O)(=O)C(F)(F)C(F)(F)C(F)(F)C(F)(F)C(F)(F)C(F)(F)C(F)(F)C(F)(F)F)CCCS(O)(=O)=O
N-SP-FHxSA	N-sulfopropyl perfluorohexanesulfonamide	C(CNS(=O)(=O)C(C(C(C(C(C(F)(F)F)(F)F)(F)F)(F)F)(F)F)(F)F)CS(=O)(=O)O
FHxSAA	Perfluorohexyl sulfonamidoacetic acid	C(C(=O)O)NS(=O)(=O)C(C(C(C(C(C(F)(F)F)(F)F)(F)F)(F)F)(F)F)(F)F
FHxSA-PrA	perfluorohexane sulfonamido propionate	N(CCC(=O)O)S(=O)(=O)C(C(C(C(C(C(F)(F)F)(F)F)(F)F)(F)F)(F)F)(F)F
MeFHxSA	N-methyl-perfluorohexanesulfonamide	CNS(=O)(=O)C(C(C(C(C(C(F)(F)F)(F)F)(F)F)(F)F)(F)F)(F)F
NMe-PFHxSAmSi	N-methyl-perfluorohexane sulfonamido sulfinate	S(O)(=O)N(C)S(=O)(=O)C(C(C(C(C(C(F)(F)F)(F)F)(F)F)(F)F)(F)F)(F)F
NMe-PFOSAmSi	N-methyl-perfluorooctane sulfonamido sulfinate	S(O)(=O)N(C)S(=O)(=O)C(C(C(C(C(C(C(C(F)(F)F)(F)F)(F)F)(F)F)(F)F)(F)F)(F)F)(F)F
U-PFHxSBC	Perfluorohexyl amido betaine unsaturated carboxylate	C[N+](C)(CCC([O-])=O)\C=C\CN(CCC(O)=O)S(=O)(=O)C(F)(F)C(F)(F)C(F)(F)C(F)(F)C(F)(F)C(F)(F)F
4:2 FTS	4:2 fluorotelomer sulfonate	C(CS(=O)(=O)O)C(C(C(C(F)(F)F)(F)F)(F)F)(F)F

6:2 FTS	6:2 fluorotelomer sulfonate	C(CS(=O)(=O)O)C(C(C(C(C(C(F)(F)F)(F)F)(F)F)(F)F)(F)F)(F)F
8:2 FTS	8:2 fluorotelomer sulfonate	C(CS(=O)(=O)O)C(C(C(C(C(C(C(C(F)(F)F)(F)F)(F)F)(F)F)(F)F)(F)F)(F)F)(F)F
6:2 FTUS	Unsaturated 6:2 fluorotelomer sulfonate	C(=CS(=O)(=O)O)C(C(C(C(C(C(F)(F)F)(F)F)(F)F)(F)F)(F)F)(F)F
8:2 FTUS	Unsaturated 8:2 fluorotelomer sulfonate	C(=CS(=O)(=O)O)C(C(C(C(C(C(C(C(F)(F)F)(F)F)(F)F)(F)F)(F)F)(F)F)(F)F)(F)F
OH-6:2 FTS	hydroxy(OH)-6:2 fluorotelomer sulfonate	C(C(O)S(=O)(=O)O)C(C(C(C(C(C(F)(F)F)(F)F)(F)F)(F)F)(F)F)(F)F
OH-8:2 FTS	hydroxy(OH)-8:2 fluorotelomer sulfonate	C(C(O)S(=O)(=O)O)C(C(C(C(C(C(C(C(F)(F)F)(F)F)(F)F)(F)F)(F)F)(F)F)(F)F)(F)F
Ketone-6:2 FTS	Ketone-6:2 fluorotelomer sulfonate	C(=O)(CS(=O)(=O)O)C(C(C(C(C(C(F)(F)F)(F)F)(F)F)(F)F)(F)F)(F)F
Ketone-8:2 FTS	Ketone-8:2 fluorotelomer sulfonate	C(=O)(CS(=O)(=O)O)C(C(C(C(C(C(C(C(F)(F)F)(F)F)(F)F)(F)F)(F)F)(F)F)(F)F)(F)F
nH-Ketone-5:2 FTS	Hydrido-ketone-5:2-fluorotelomer sulfonate	C(=O)(CC(C(C(C(C(F)F)(F)F)(F)F)(F)F)(F)F)S(=O)(=O)O
nH-ketone 7:2 FTS	Hydrido-ketone-7:2-fluorotelomer sulfonate	C(=O)(CC(C(C(C(C(C(C(F)F)(F)F)(F)F)(F)F)(F)F)(F)F)(F)F)S(=O)(=O)O
6:2 FTSO2PA (n=3)	6:2 fluorotelomersulfonyl propanoic acid	FC(F)(F)C(F)(F)C(F)(F)C(F)(F)C(F)(F)C(F)(F)CCS(CCC(O)=O)(=O)=O
8:2 FTSO2PA (n=4)	8:2 fluorotelomersulfonyl propanoic acid	C(CS(=O)(=O)CCC(C(C(C(C(C(C(C(F)(F)F)(F)F)(F)F)(F)F)(F)F)(F)F)(F)F)(F)F)C(=O)O
n:2 FTSO2PAASAs n=3	6:2 fluorotelomer mercaptoalkylamido sulfonate sulfone	CC(C)(CS(=O)(=O)O)NC(=O)CCS(=O)(=O)CCC(C(C(C(C(C(F)(F)F)(F)F)(F)F)(F)F)(F)F)(F)F
n:2 FTSO2PAASAs n=4	8:2 fluorotelomer mercaptoalkylamido sulfonate sulfone	CC(C)(CS(=O)(=O)O)NC(=O)CCS(=O)(=O)CCC(C(C(C(C(C(C(C(F)(F)F)(F)F)(F)F)(F)F)(F)F)(F)F)(F)F)(F)F
6:2 FTSASA	6:2 Fluorotelomer sulfinyl amido sulfonic acid	CC(C)(CS(=O)(=O)O)NC(=O)CCS(=O)CCC(C(C(C(C(C(F)(F)F)(F)F)(F)F)(F)F)(F)F)(F)F
6:2 diPAP	Bis[2-(perfluorohexyl)ethyl] Phosphate	C(COP(=O)(O)OCCC(C(C(C(C(C(F)(F)F)(F)F)(F)F)(F)F)(F)F)(F)F)C(C(C(C(C(C(F)(F)F)(F)F)(F)F)(F)F)(F)F)(F)F
6:2 FTSAm	6:2 fluorotelomer sulfonamide	NS(=O)(=O)CCC(F)(F)C(F)(F)C(F)(F)C(F)(F)C(F)(F)C(F)(F)F
6:2 FTSi	6:2 fluorotelomer sulfinate	C(CS(=O)O)C(C(C(C(C(C(F)(F)F)(F)F)(F)F)(F)F)(F)F)(F)F
5:3 FTUCA	5:3 fluorotelomer unsaturated carboxylic acid	C(=CC(C(C(C(C(F)(F)F)(F)F)(F)F)(F)F)(F)F)C(=O)O
OH-PFHxAA	Perfluorohexyl-2-hydroxyacetic acid	C(C(=O)O)(O)C(C(C(C(C(C(F)(F)F)(F)F)(F)F)(F)F)(F)F)(F)F

Table S7: List of PFAS classes and homologues detected in natural foam, surface waters (SW1–SW4; n = 4), and groundwater wells ($n_{1.5\,km}$ = 3; $n_{3.5\,km}$ = 4) with ∑17 PFAS > 30 ng/L, as well as documented detections at the former firefighter training facility (FFTA).[8]

#Class	(C_n) Detected in:				Liu et al.[8] (FFTA)
	Natural foam	Surface water	Well (1.5 km)	Well (3.5 km)	
PFCA	8–11	4–12	4–10	4–10	3–12
PFSA	8–10	3–10	3–8	3–8	3–10
FASA	6,8	3–8	3–6	4,7,8	3–8
n:2 FTS	8	4,6,8	6	×	4,6,8
n:2 FTSAm	×	6	×	6	-
n:2 FTUSA	8††	6,8††	6	×	-
OH-n:2 FTSA	×	6,8	6	×	×
Ketone-n:2 FTSA	8††	6,8††	6	6	-
n:3 FTUCA	×	5	×	×	-
H-PFSA	×	8	8	8	×
H-UPFSA	×	4,6	×	×	×
Ketone-PFSA	×	7	×	×	×
U-PFSA	×	6,7	×	×	×
Cl-PFSA	×	7	×	×	×
O-PFSA	×	7,8	×	×	×
PFACHS	×	2	2	2	2
FASA-PrA	×	6††	×	×	-
MeFASA	×	6	×	×	-
N-SP-FASA	×	6	×	×	6
n:2 FTSO2PA	×	6,8	×	×	6,8
OH-PFAAA	×	6	×	×	-
FASAA	×	6	×	×	×
Sn:2 diPAP	×	6	×	×	-
F5S-PFAS	×	8	×	×	8
n:2 FTSO2PAASA	×	6,8	×	×	6,8
n:2 FTSASA	×	6	×	×	6
N-SPAMP-FASA	×	6	×	×	6
N-SPAMP-FASAA	×	6	×	×	×
N-SPAMP-FASAPS	×	6,8	×	×	×
n:2 FTSi	×	6*	×	×	-
U-PFASBC	×	6*	×	×	-
nH-Ketone-n:2 FTSA	×	5††,7††	5††	5††	-
NMe-PFASAmSi	×	6††,8††	8††	8††	-

*"**n**": number of perfluorinated carbons (see Fig. 1b); "×": Not detected; "**-**": Not characterized at FFTA; "**††**": Novel PFAS (Schymanski level 2 and 3a), "*****": Novel PFAS (Schymanski level 3b)[6,7].*

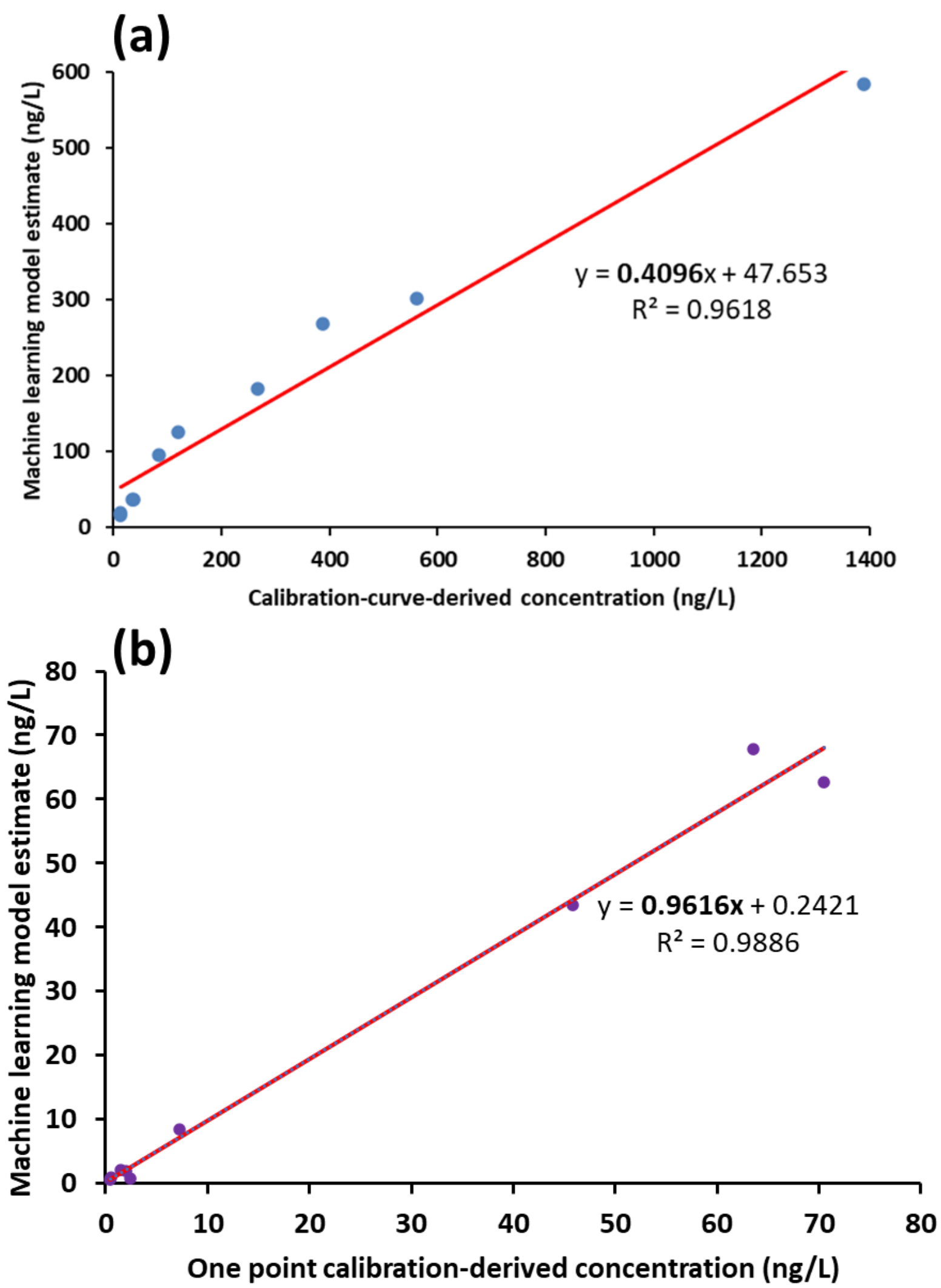

Figure S1: Scatterplot comparing (a) the sum concentration of 21 targeted PFAS (exc. PFBA and PFPeA) estimated using a 5-point calibration curve versus a machine-learning model prediction[9]; and (b) the sum concentration of 49 non-targeted PFAS (*Schymanski level 2, 3a, and 3b*) estimated using a single-point calibration ($^{13}C_4$-PFOS) and the machine learning model[9] in nine representative water samples, including 3 surface water and 6 groundwater samples.

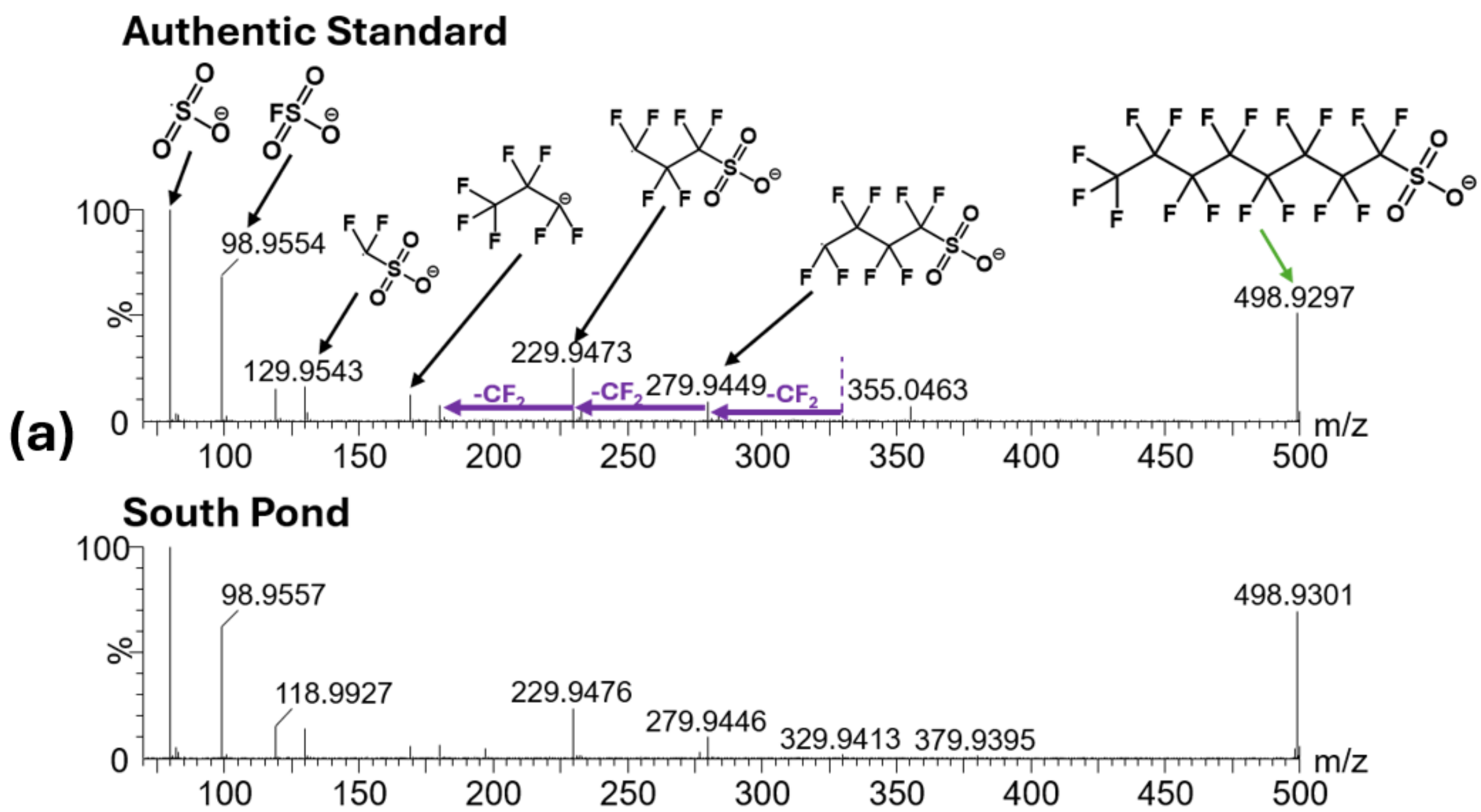

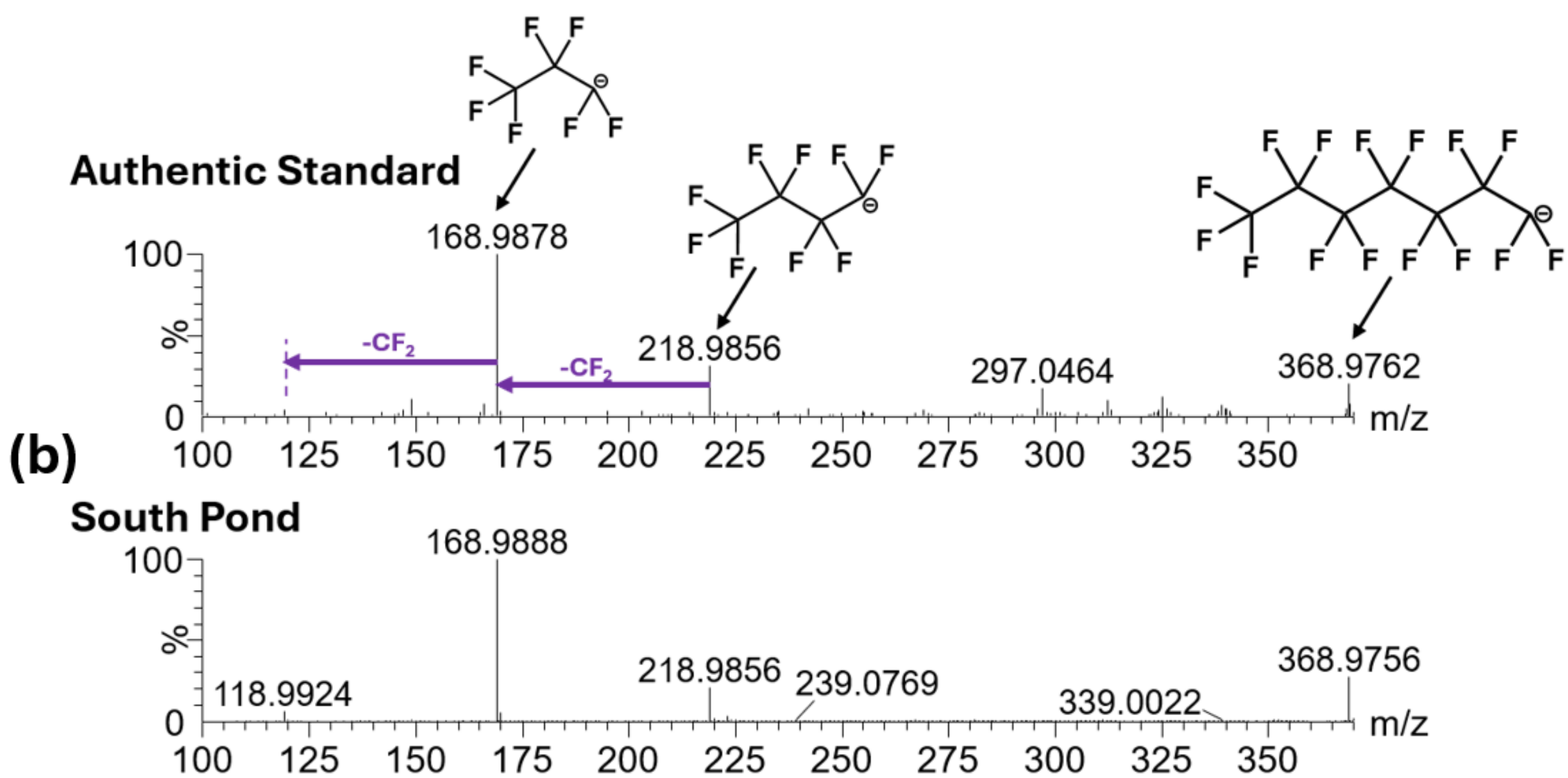

Figure S2: Annotated MS² CID spectra of (a) **PFOS** and (b) **PFOA**, comparing an authentic analytical standard (top) against a surface water sample collected from South Pond (bottom). Overlapping fragment ions illustrate structural similarity. Collision Energy (CE) = 35 ± 15V.

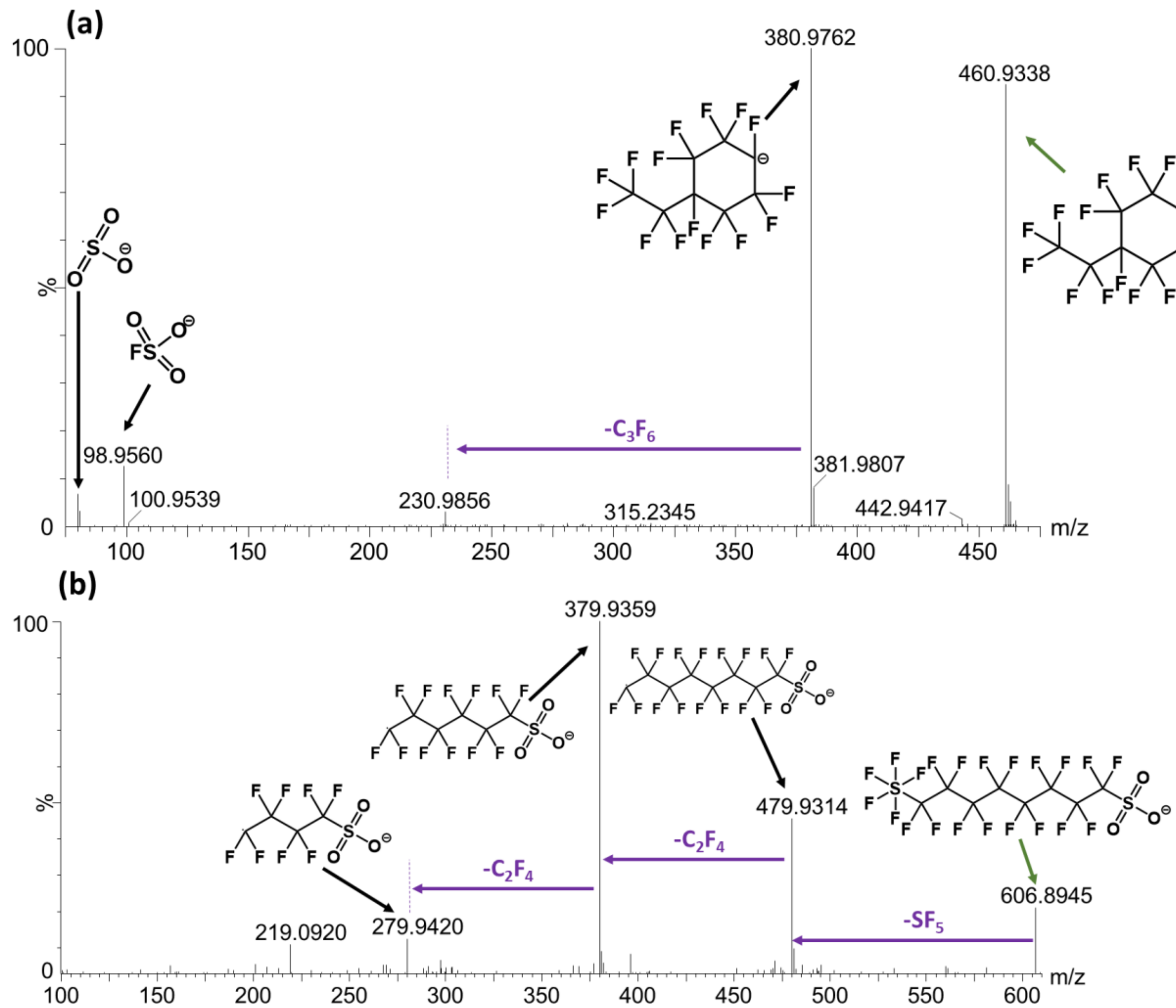

Figure S3: Annotated MS² CID spectra of unknown features (a) **PFECHS**[10] (m/z 460.9338, *level 2*); and (b) **F5S-PFOS**[11] (m/z 606.8945, *level 2*). Collision Energy (CE) = 35 ± 15V.

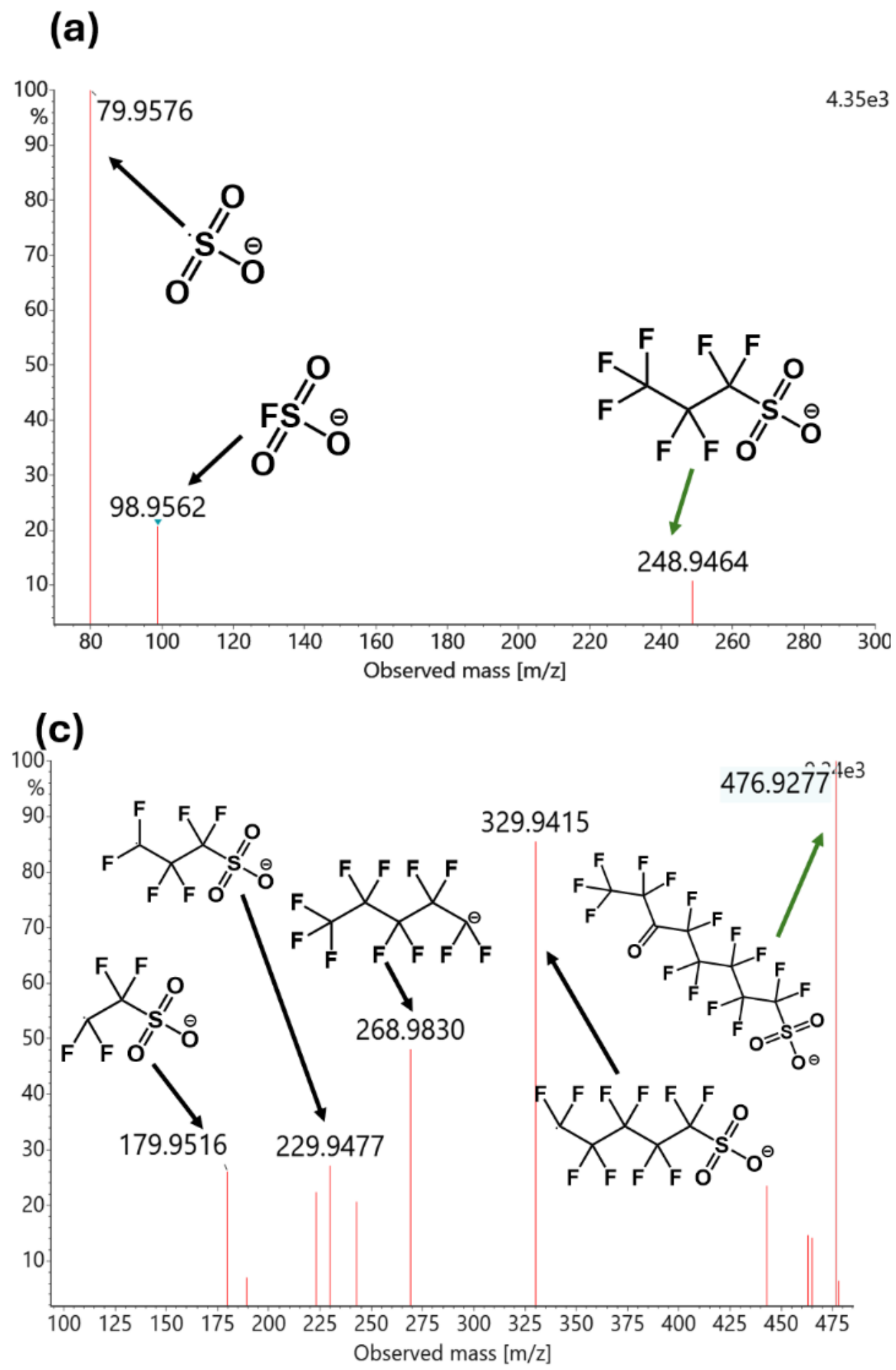

Figure S4: Annotated high-energy spectra of unknown features (a) **PFPrS**[10] (m/z 248.9461, *level 2*), (b) **H-PFOS**[11] (m/z 480.9401, *level 3a*), (c) **Ketone-PFOS**[10] (m/z 476.9283, *level 3a*), (d) **H-U-PFOS**[11] (m/z 442.9439, *level 3a*). Collision energy (CE) = 20 – 50V

**The hydrogen atom (H) in H-PFOS and H-U-PFOS could not be established by MS/MS alone, hence the Level 3a assignment*

.

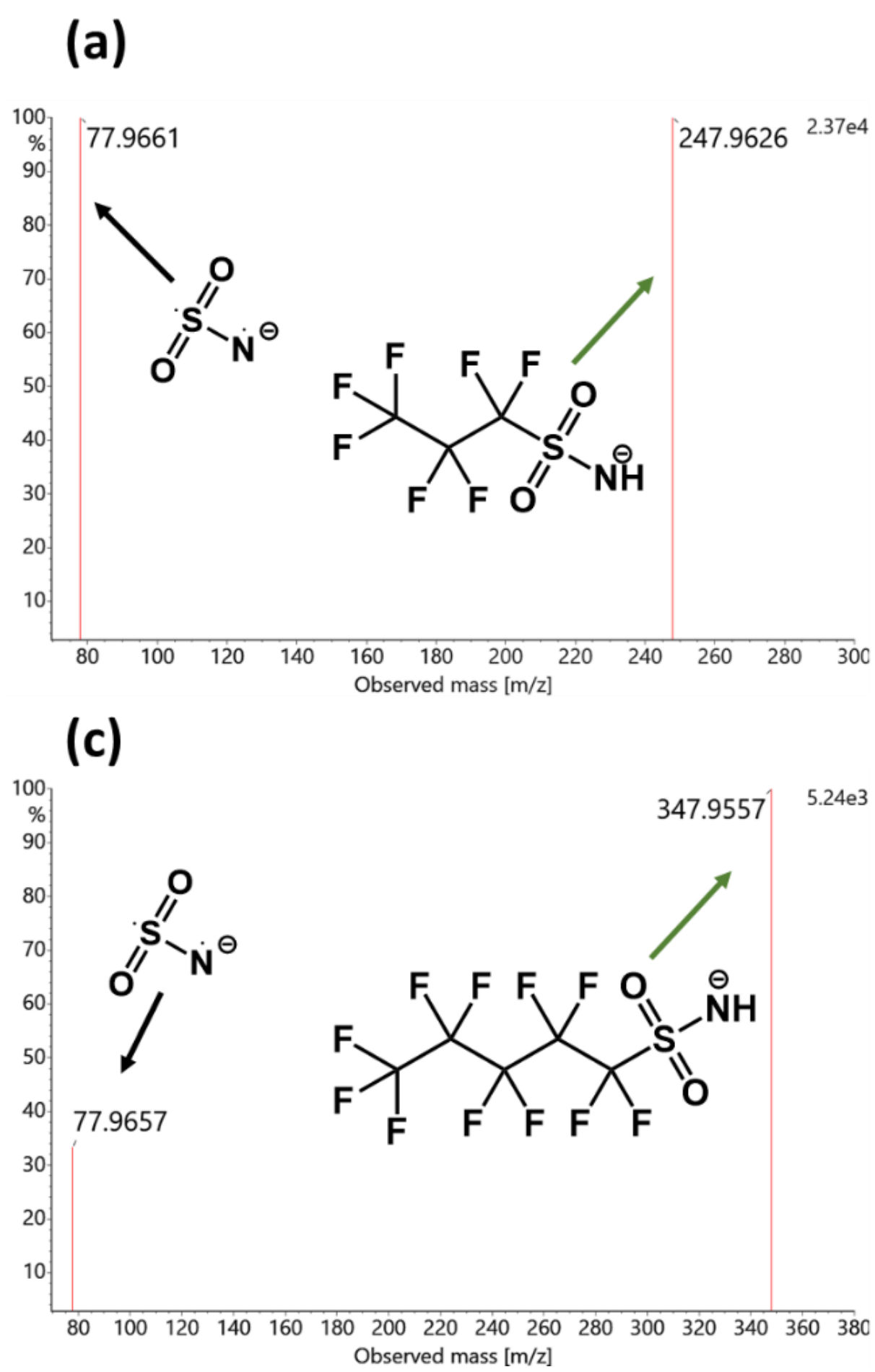

Figure S5: Annotated high-energy spectra of unknown features (a) **FPrSA**[12] (m/z 247.9626, *level 2*), (b) **FBSA**[12] (m/z 297.9591, *level 2*), (c) **FPeSA**[12] (m/z 347.9562, *level 2*), (d) **FHxSA**[12] (m/z 397.9528, *level 2*). Collision energy (CE) = 20 – 50V.

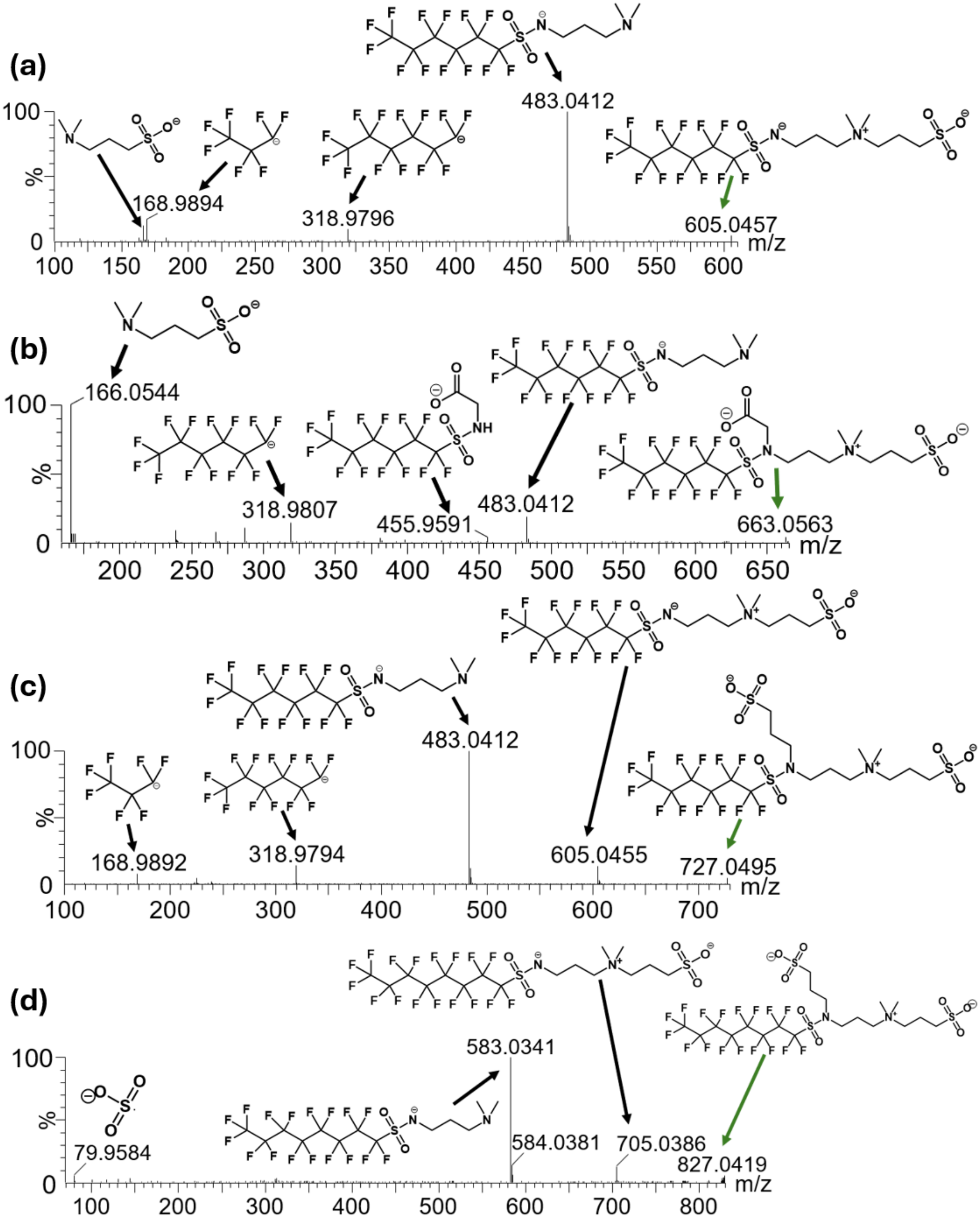

Figure S6: Annotated MS[2] CID spectra of unknown features (a) **SPAMP-FHxSA**[11] (m/z 605.0457, *level 2*), (b) **SPAMP-FHxSAA**[11] (m/z 663.0510, *level 2*), (c) **SPAMP-FHxSAPS**[11] (m/z 727.0495, *level 2*) (d) **SPAMP-FOSAPS**[11] (m/z 827.0419, *level 2*). Collision energy (CE) = 35 ± 15V.

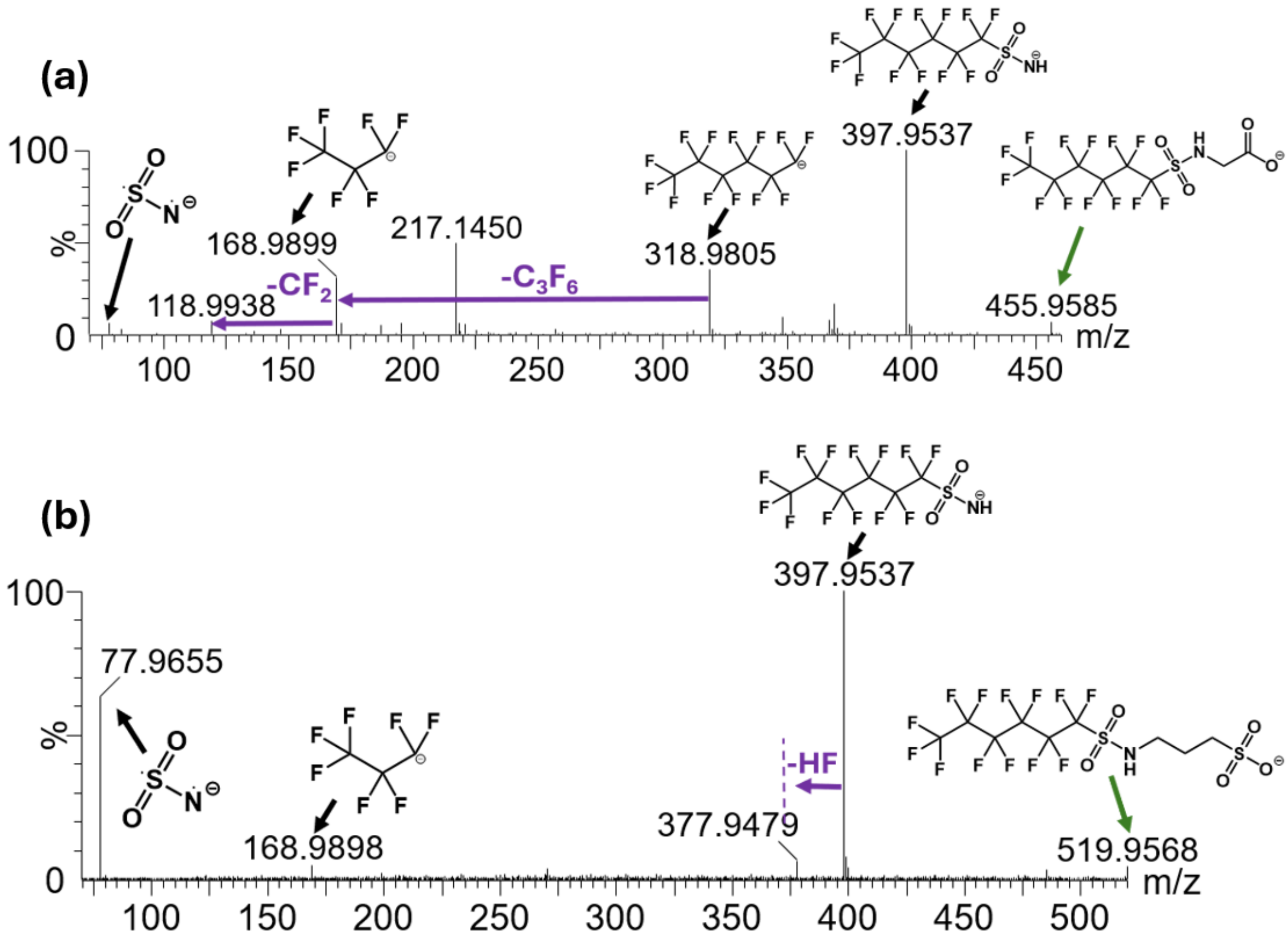

Figure S7: Annotated MS^2 CID spectra of unknown features (a) **FHxSAA**[10,11] (m/z 455.9585, *level 2*), (b) **N-SP-FHxSA**[11] (m/z 519.9568, *level 2*). Collision energy (CE) = 35 ± 15V.

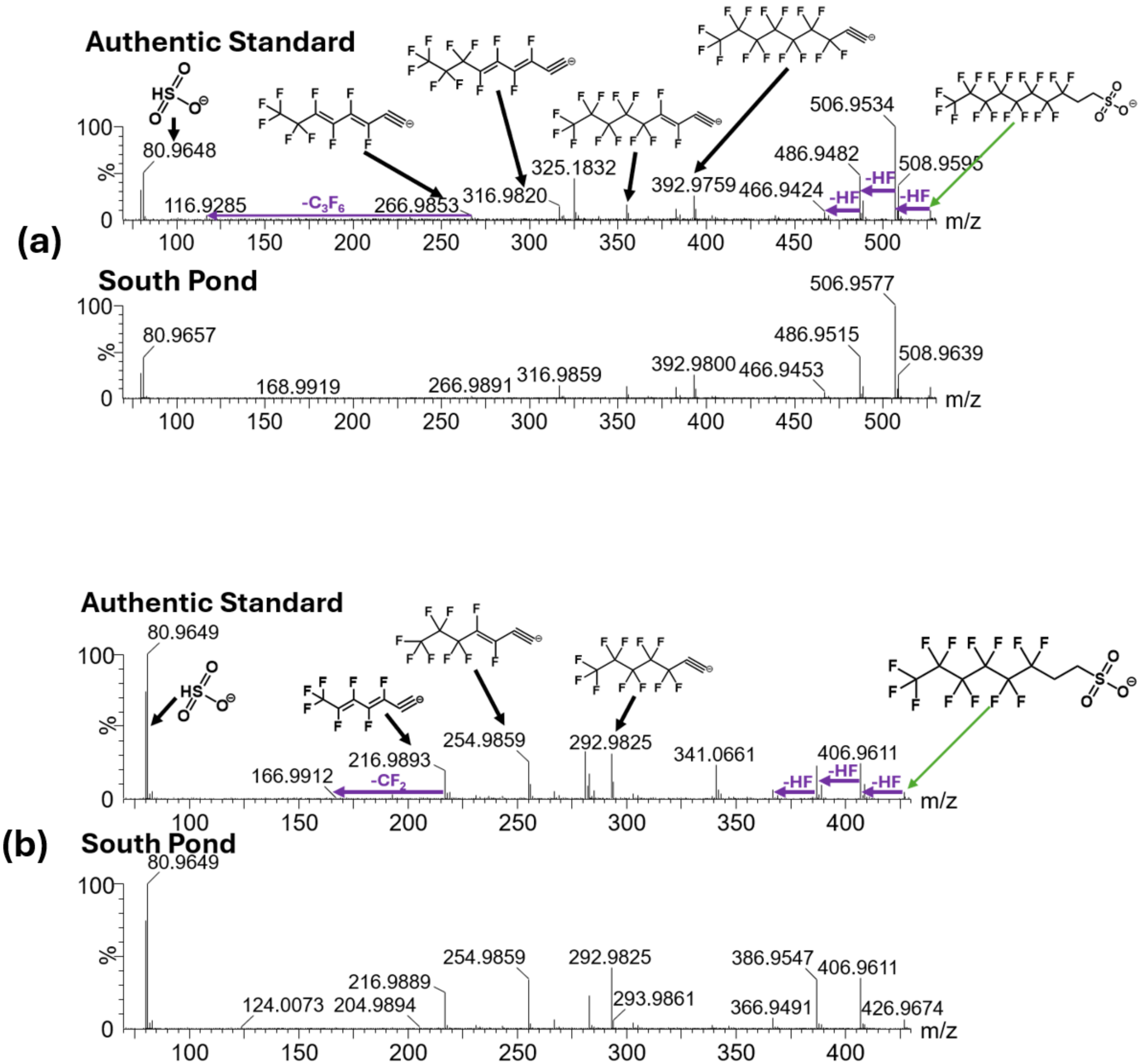

Figure S8: Annotated MS[2] CID spectra of (a) **8:2 FTS** and (b) **6:2 FTS**, comparing an authentic analytical standard (top) against a surface water sample collected from South Pond (bottom). Overlapping fragment ions illustrate structural similarity. Collision Energy (CE) = 35 ± 15V.

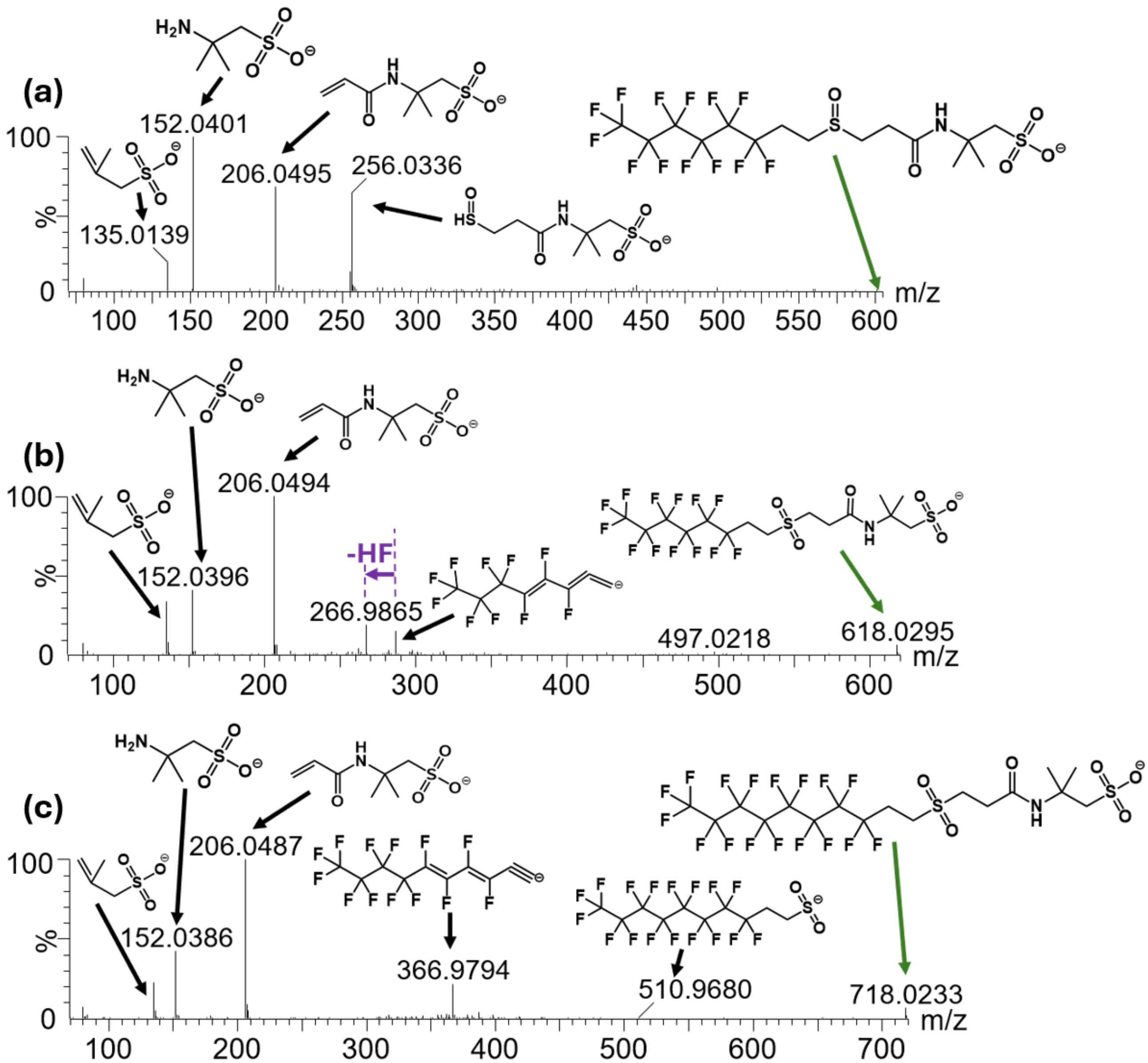

Figure S9: Annotated MS^2 CID spectra of unknown features (a) **6:2 FTSASA**[10] (m/z 602.0341, *level 2*), (b) **n:2 FTSO2PAASAs, n = 3**[10,11] (m/z 618.0295, *level 2*), (c) **n:2 FTSO2PAASAs, n = 4**[10,11] (m/z 718.0233, *level 2*). Collision energy (CE) = 35 ± 15V.

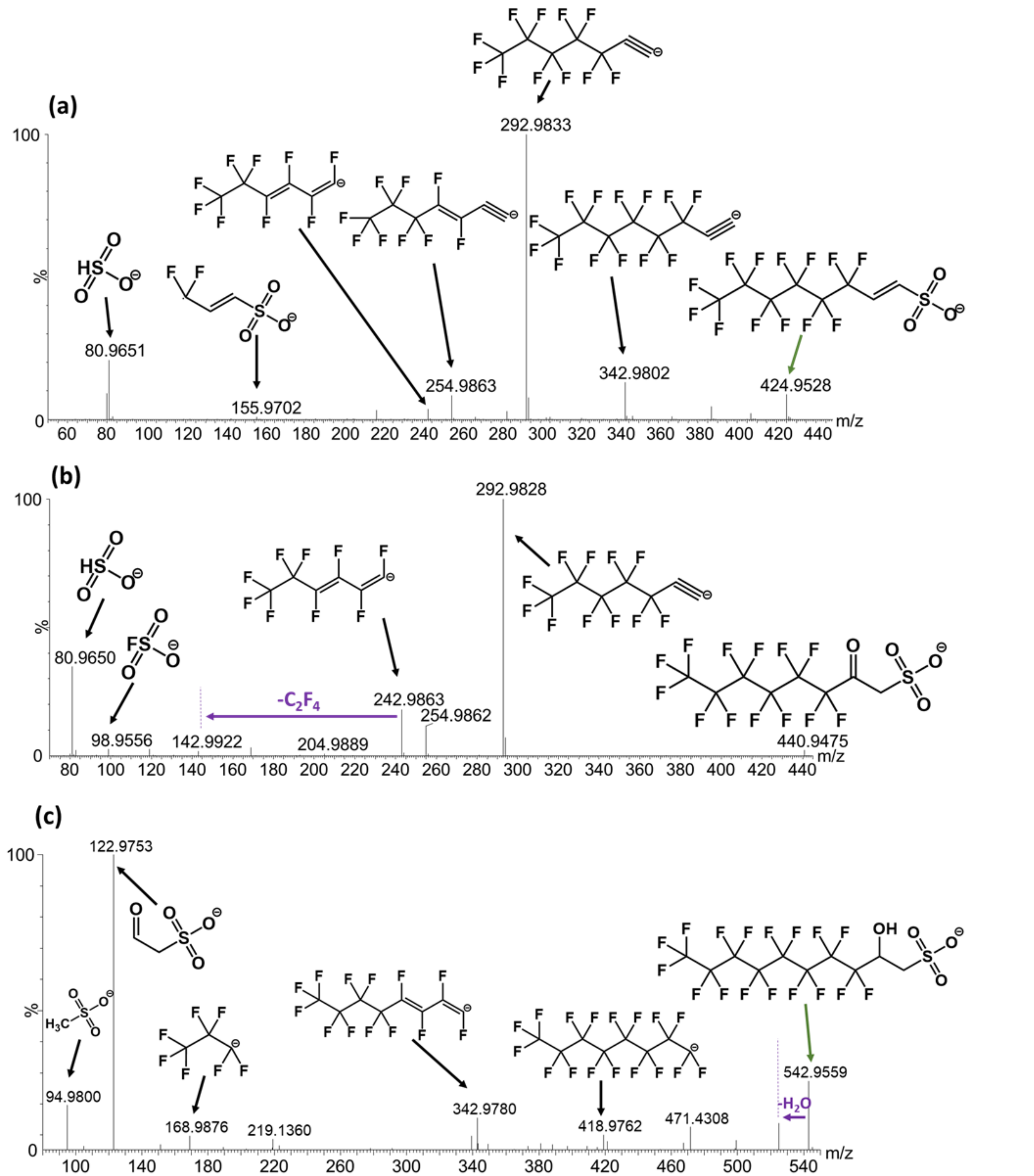

Figure S10: Annotated MS^2 CID spectra of unknown features (a) **6:2 FTUS**[11,13] (m/z 424.9528, *level 2*), (b) **Ketone-6:2 FTS**[13] (m/z 440.9475, *level 3a*), (c) **OH-8:2 FTS**[11] (m/z 542.9561, *level 3a*), MS^2 CID Spectrum. Collision Energy (CE) = 35 ± 15V.

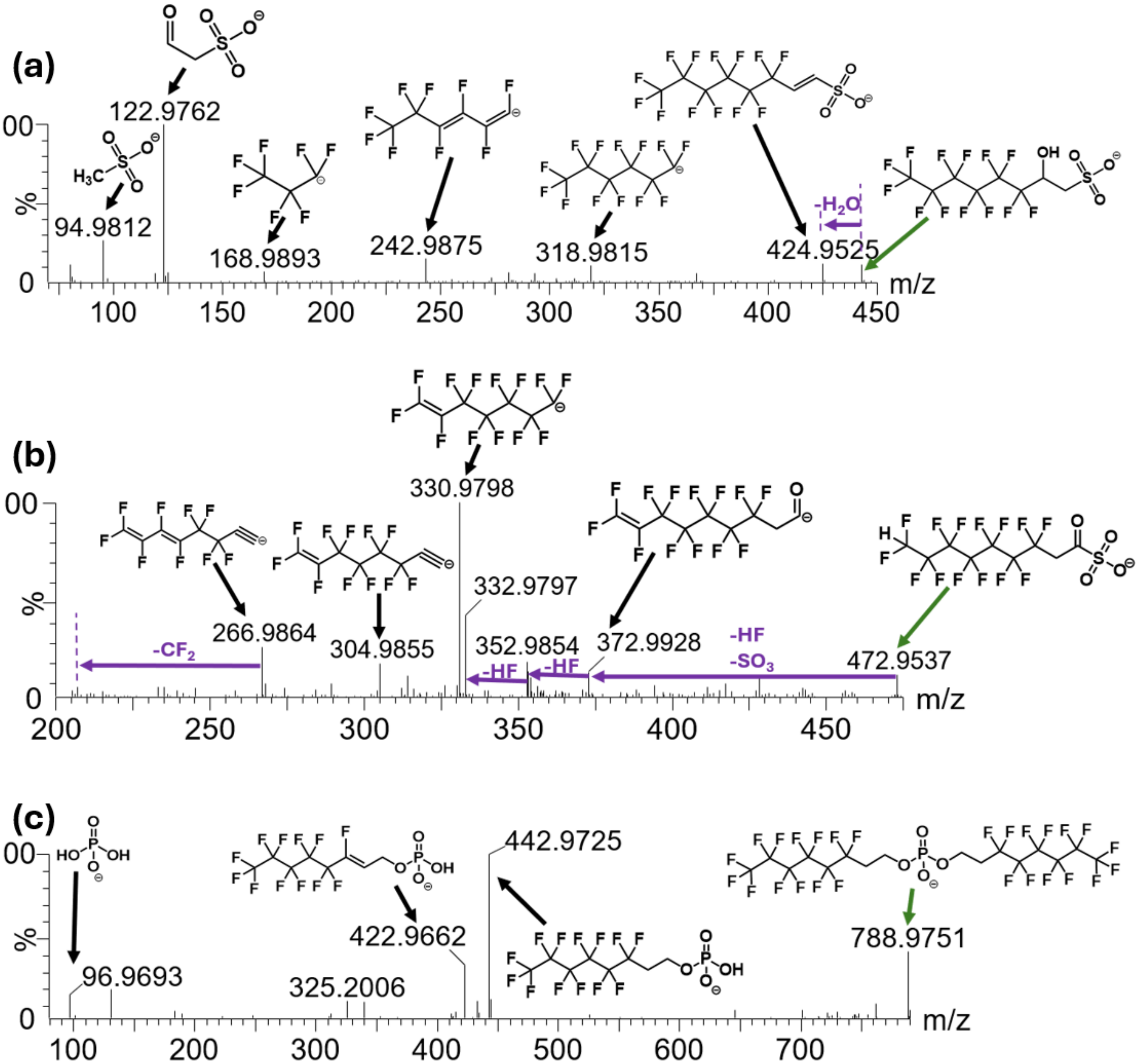

Figure S11: Annotated MS^2 CID spectra of unknown features (a) **OH-6:2 FTS**[13] (m/z 442.9630, *level 3a*), (b) **nH-Ketone-7:2 FTS** (m/z 472.9537, *level 3a*), (c) **6:2 diPAP**[14] (m/z 788.9751, *level 2*). Collision energy (CE) = 35 ± 15V.

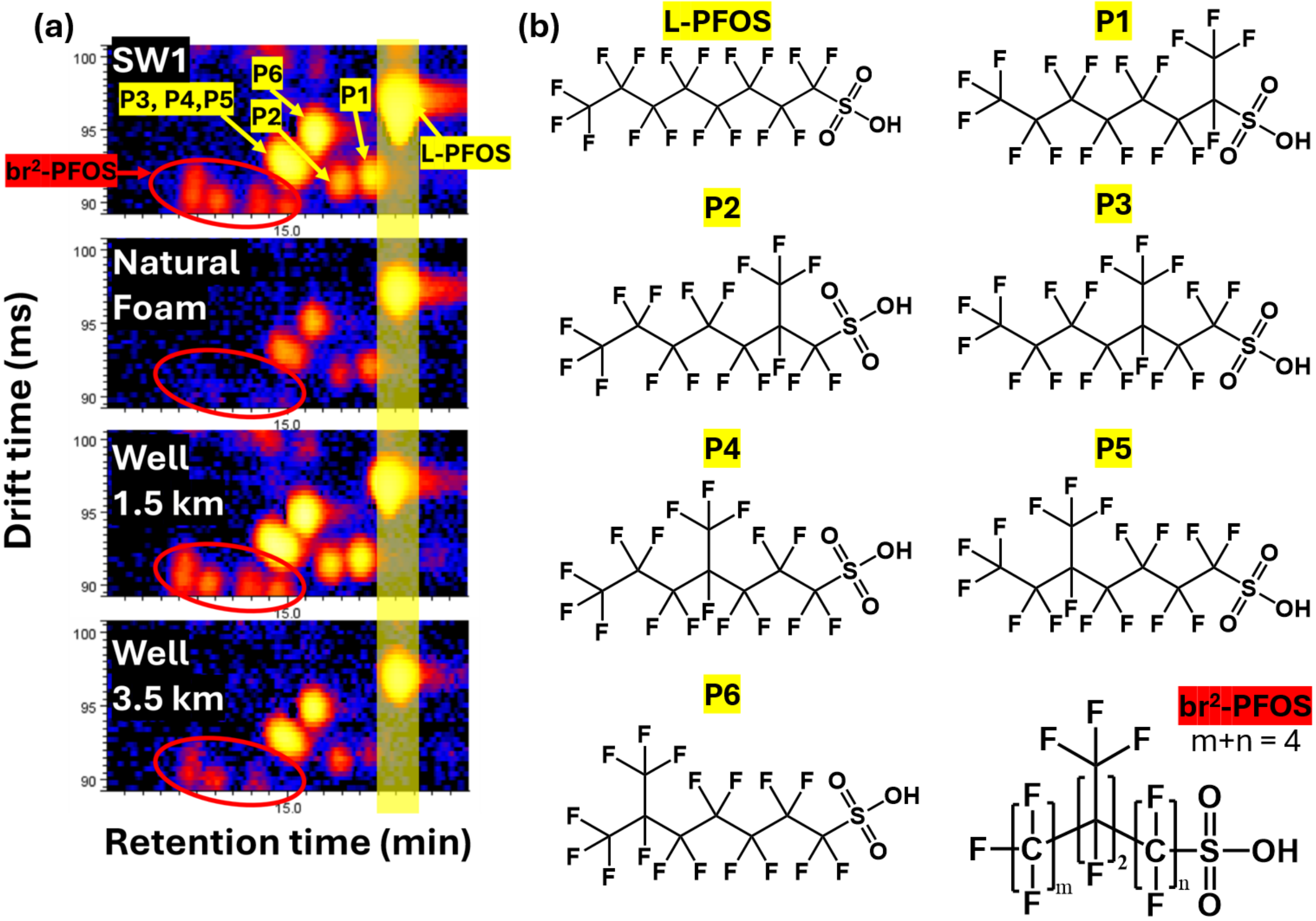

Figure S12: Heatmap showing RT (min) vs. Drift time (ms) of perfluorooctane sulfonate (PFOS) isomers resolved under a 78 ms multipass cIMS separation, in South Pond (SW1), natural foam, and groundwater wells located 1.5 km and 3.5 km away from the firefighter training facility with ∑17 PFAS > 100 ng/L.

**Annotations are based on Wijayahena et al.[15] including,*

L-PFOS: *Perfluorooctane sulfonic acid*

P1: *Perfluoro-1-methylheptane sulfonate*

P2: *Perfluoro-2-methylheptane sulfonate*

P3: *Perfluoro-3-methylheptane sulfonate*

P4: *Perfluoro-4-methylheptane sulfonate*

P5: *Perfluoro-5-methylheptane sulfonate*

P6: *Perfluoro-6-methylheptane sulfonate*

br²-PFOS: *Perfluorodimethylhexane sulfonate isomers*

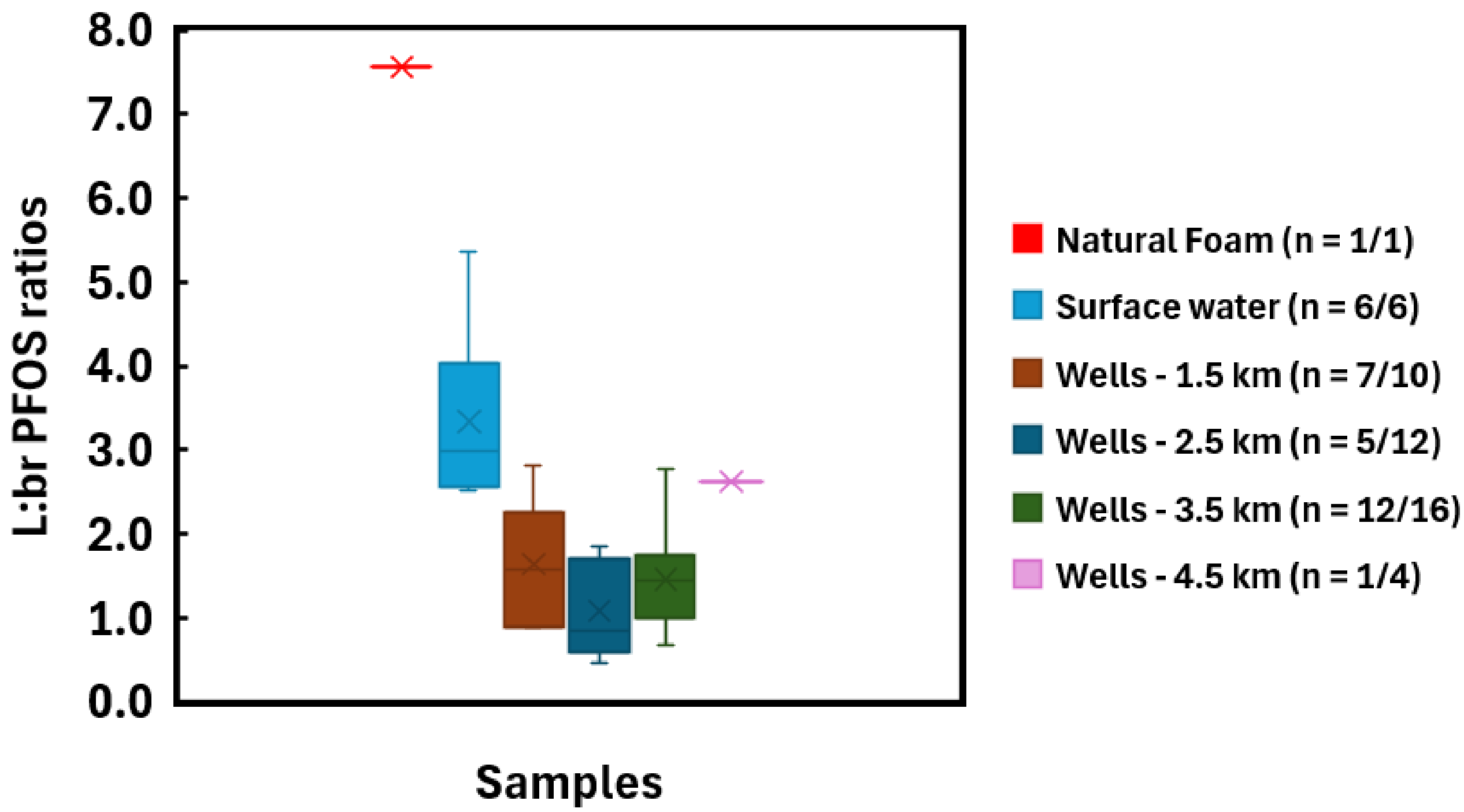

Figure S13: Boxplots comparing the **linear-to-branched (L:br) PFOS isomer ratios** in surface water sites (SW1–SW6, n = 6) and groundwater wells (n = 25) between 1.5 km and 4.5 km from the former firefighter training area (FFTA), containing detectable concentrations of PFOS.

**Statistical significance was assessed using two-sample t-tests. Surface water sites differed significantly from groundwater wells located 1.5–3.5 km from the source ($p < 0.05$), whereas no significant differences were observed among groundwater distance groups ($p > 0.05$). Natural foam and the 4.5 km groundwater well were excluded from statistical analysis because only a single data point was available for each.*

References

(1) Wu, Z.; Gao, W.; Phelps, M. A.; Wu, D.; Miller, D. D.; Dalton, J. T. Favorable Effects of Weak Acids on Negative-Ion Electrospray Ionization Mass Spectrometry. *Anal. Chem.* **2004**, *76* (3), 839–847. https://doi.org/10.1021/ac0351670.

(2) Labine, L. M.; Oliveira Pereira, E. A.; Kleywegt, S.; Jobst, K. J.; Simpson, A. J.; Simpson, M. J. Comparison of Sub-Lethal Metabolic Perturbations of Select Legacy and Novel Perfluorinated Alkyl Substances (PFAS) in Daphnia Magna. *Environmental Research* **2022**, *212*, 113582. https://doi.org/10.1016/j.envres.2022.113582.

(3) Critch-Doran, O.; Jenkins, K.; Hashemihedeshi, M.; Mommers, A. A.; Green, M. K.; Dorman, F. L.; Jobst, K. J. Toward Part-per-Million Precision in the Determination of an Ion's Collision Cross Section Using Multipass Cyclic Ion Mobility. *J. Am. Soc. Mass Spectrom.* **2024**, *35* (4), 775–783. https://doi.org/10.1021/jasms.4c00003.

(4) Zhang, H.; Luo, M.; Wang, H.; Ren, F.; Yin, Y.; Zhu, Z.-J. AllCCS2: Curation of Ion Mobility Collision Cross-Section Atlas for Small Molecules Using Comprehensive Molecular Representations. *Anal. Chem.* **2023**, *95* (37), 13913–13921. https://doi.org/10.1021/acs.analchem.3c02267.

(5) Joseph, K.; Boatman, A.; Dodds, J.; Kirkwood Donelson, K.; Ryan, J.; Zhang, J.; Thiessen, P.; Bolton, E.; Valdiviezo, A.; Sapozhnikova, Y.; Rusyn, I.; Schymanski, E.; Baker, E. Dataset for "Multidimensional Library for the Improved Identification of per- and Polyfluoroalkyl Substances (PFAS)," 2024. https://doi.org/10.5281/ZENODO.14341321.

(6) Schymanski, E. L.; Jeon, J.; Gulde, R.; Fenner, K.; Ruff, M.; Singer, H. P.; Hollender, J. Identifying Small Molecules via High Resolution Mass Spectrometry: Communicating Confidence. *Environ. Sci. Technol.* **2014**, *48* (4), 2097–2098. https://doi.org/10.1021/es5002105.

(7) Boatman, A. K.; Chappel, J. R.; Kirkwood-Donelson, K. I.; Fleming, J. F.; Reif, D. M.; Schymanski, E. L.; Rager, J. E.; Baker, E. S. Updated Guidance for Communicating PFAS Identification Confidence with Ion Mobility Spectrometry. January 28, 2025. https://doi.org/10.1101/2025.01.27.634925.

(8) Liu, M.; Munoz, G.; Vo Duy, S.; Sauvé, S.; Liu, J. Per- and Polyfluoroalkyl Substances in Contaminated Soil and Groundwater at Airports: A Canadian Case Study. *Environ. Sci. Technol.* **2022**, *56* (2), 885–895. https://doi.org/10.1021/acs.est.1c04798.

(9) Liigand, J.; Wang, T.; Kellogg, J.; Smedsgaard, J.; Cech, N.; Kruve, A. Quantification for Non-Targeted LC/MS Screening without Standard Substances. *Sci Rep* **2020**, *10* (1), 5808. https://doi.org/10.1038/s41598-020-62573-z.

(10) Wu, C.; Wang, Q.; Chen, H.; Li, M. Rapid Quantitative Analysis and Suspect Screening of Per-and Polyfluorinated Alkyl Substances (PFASs) in Aqueous Film-Forming Foams (AFFFs) and Municipal Wastewater Samples by Nano-ESI-HRMS. *Water Research* **2022**, *219*, 118542. https://doi.org/10.1016/j.watres.2022.118542.

(11) Barzen-Hanson, K. A.; Roberts, S. C.; Choyke, S.; Oetjen, K.; McAlees, A.; Riddell, N.; McCrindle, R.; Ferguson, P. L.; Higgins, C. P.; Field, J. A. Discovery of 40 Classes of Per- and Polyfluoroalkyl Substances in Historical Aqueous Film-Forming Foams (AFFFs) and AFFF-Impacted Groundwater. *Environ. Sci. Technol.* **2017**, *51* (4), 2047–2057. https://doi.org/10.1021/acs.est.6b05843.

(12) Dewapriya, P.; Nilsson, S.; Ghorbani Gorji, S.; O'Brien, J. W.; Bräunig, J.; Gómez Ramos, M. J.; Donaldson, E.; Samanipour, S.; Martin, J. W.; Mueller, J. F.; Kaserzon, S. L.; Thomas, K. V. Novel Per- and Polyfluoroalkyl Substances Discovered in Cattle Exposed to AFFF-Impacted Groundwater. *Environ. Sci. Technol.* **2023**, *57* (36), 13635–13645. https://doi.org/10.1021/acs.est.3c03852.

(13) Fang, B.; Zhang, Y.; Chen, H.; Qiao, B.; Yu, H.; Zhao, M.; Gao, M.; Li, X.; Yao, Y.; Zhu, L.; Sun, H. Stability and Biotransformation of 6:2 Fluorotelomer Sulfonic Acid, Sulfonamide Amine Oxide, and Sulfonamide Alkylbetaine in Aerobic Sludge. *Environ. Sci. Technol.* **2024**, *58* (5), 2446–2457. https://doi.org/10.1021/acs.est.3c05506.

(14) Bugsel, B.; Bauer, R.; Herrmann, F.; Maier, M. E.; Zwiener, C. LC-HRMS Screening of per- and Polyfluorinated Alkyl Substances (PFAS) in Impregnated Paper Samples and Contaminated Soils. *Anal Bioanal Chem* **2022**, *414* (3), 1217–1225. https://doi.org/10.1007/s00216-021-03463-9.

(15) Wijayahena, M. K.; Kachangoon, R.; Witmer, C.; Wallace, J. S.; Vichapong, J.; Aga, D. S. PFAS Isomers Matter: Distribution Patterns of Linear and Branched PFOS and PFOA in Consumed Fish Revealed by Cyclic Ion Mobility Separation. *J. Agric. Food Chem.* **2025**, *73* (41), 25967–25977. https://doi.org/10.1021/acs.jafc.5c08608.
